# Microbial transglutaminase in food biotechnology: from biochemical mechanisms to industrial applications

**DOI:** 10.1007/s00253-025-13697-7

**Published:** 2026-01-24

**Authors:** Marek Kieliszek

**Affiliations:** Department of Food Biotechnology and Microbiology, Institute of Food Sciences, Warsaw University of Life Sciences—SGGW, Nowoursynowska 159 C, Warsaw, 02-776 Poland

**Keywords:** Transglutaminase, MTG, Enzyme, Cross-linked, Protein modification, Food industry

## Abstract

**Abstract:**

Microbial transglutaminase (mTG) is an enzyme produced by actinomycetes, predominantly by filamentous bacteria belonging to the genus *Streptomyces*. These microorganisms are the best-studied and most microbiologically exploited natural producers of TG. It catalyzes the formation of isopeptide bonds between glutamine and lysine residues in proteins, which alters the protein’s structure and functionality. This enzymatic activity is widely applied in the food industry, where it is particularly useful in modifying the texture, binding properties, and overall quality of protein products. The primary application of mTG in food production lies in its ability to mimic the functional properties of gluten, making it valuable in the creation of gluten-free products. By facilitating the binding of various ingredients such as starches and plant proteins, mTG helps produce products with similar texture and elasticity to those made with gluten, which is crucial for individuals with celiac disease or gluten intolerance. Beyond gluten-free applications, mTG is also employed in the production of meat substitutes, where it enhances the texture and cohesion of plant-based ingredients, as well as in bakery and confectionery products. Additionally, mTG is utilized in the development of organic materials, such as microcapsules and enzymatic carriers, owing to its calcium-independent activity, broad substrate specificity, and high stability across a wide range of pH and temperatures. The properties of this enzyme can be successfully used in the biotechnology industry. Looking forward, mTG is expected to play a significant role in the expansion of functional foods, plant-based diets, and advanced biomaterials. However, there are growing concerns about its safety, particularly regarding potential immunogenic reactions and its long-term impact on consumer health. To mitigate these risks, further research on the biological effects of mTG is essential, especially in the context of its microbial origin, molecular structure, and interaction with human proteins. Additionally, stricter regulations and clear labeling of products containing mTG will be necessary to ensure consumer safety and confidence in its widespread use.

**Key points:**

• *Microbial transglutaminase is produced by actinomycetes via fermentation.*

• *mTG is a microbial enzyme that modifies protein structure and functionality.*

• *mTG is key to future functional foods.*

• *Labeling of mTG in foods is vital for consumer trust and safety.*

• *Enzyme mTG boosts cohesion in meat substitutes and bakery goods.*

## Introduction

**Fig. 1:**
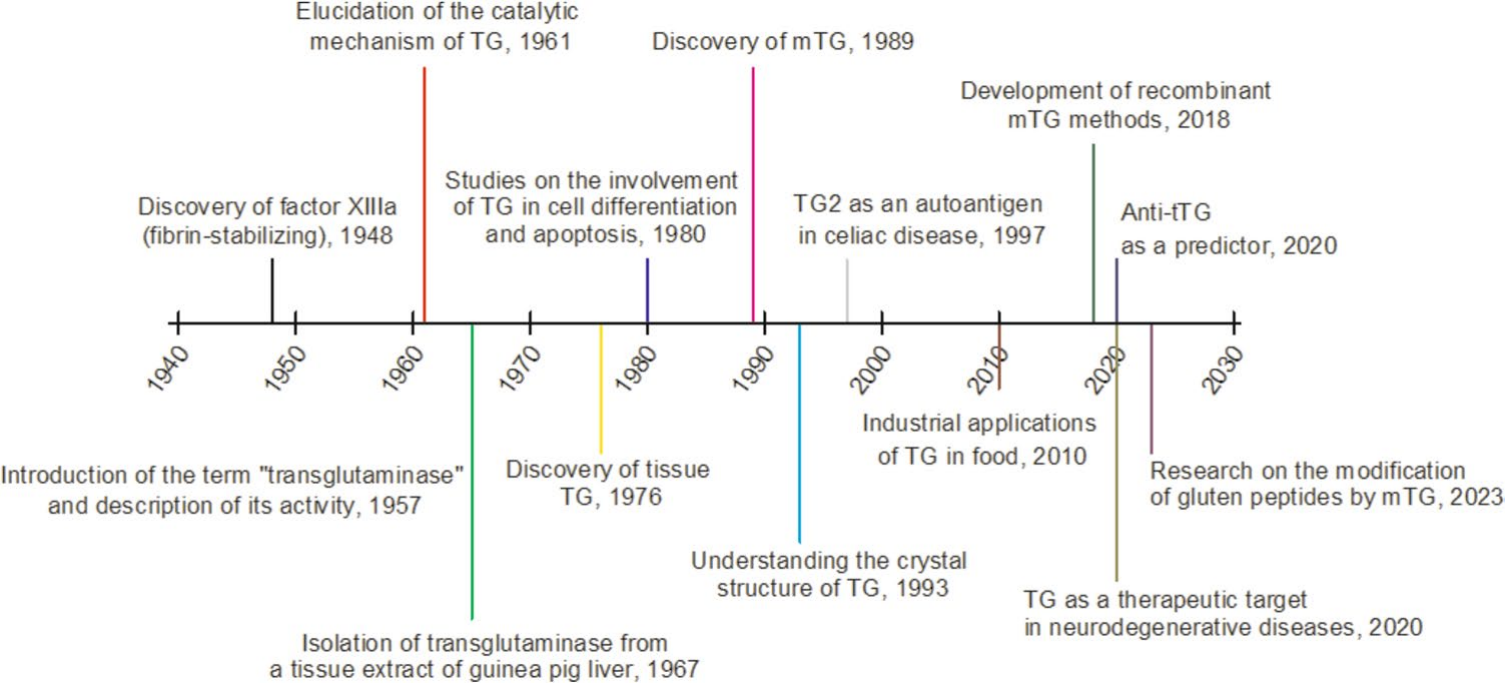
Historical outline of transglutaminase research

Transglutaminase (TG) was first observed in the 1950 s (Yao et al. 2024). This enzyme is widely distributed in nature. It can be obtained from the tissues and body fluids of fish and mammals, as well as from bacteria and plants. In 1957, Sarkar, Clarke, and Waelsh described the activity of this enzyme in tissues, primarily in the liver (Delgado and Johnson 2024). This discovery inspired further research. In 1965, the first successful isolation of transglutaminase was performed from a tissue extract of guinea pig liver (Folk and Il Chung 1985). Guinea pig liver was the most frequently chosen source of tissue transglutaminase due to its abundance of this enzyme. Gillet et al. (2004) report that 25 mg of transglutaminase, with an activity of approximately 18 U/mg, can be obtained from 140 g of guinea pig liver. Subsequent research analyzed various sources of transglutaminases, focusing on samples obtained from blood plasma (including the so-called factor XIII) (Traub et al. 2024), and skin (Kim et al. 1990). These experiments demonstrated that transglutaminases play an essential role in several biological processes, such as repairing tissue damage, regulating blood coagulation, and mechanisms related to cell differentiation (Tatsukawa et al. 2016). Transglutaminase occurs in the human body in many isoforms (Bauer et al. 2025). These include TG1 and TG2, an 80 kDa tissue transglutaminase (Yao et al. 2024), consisting of an N-terminal part, a catalytic center, and two C-terminal ends (Sadowska and Diowksz 2016). It is worth noting that biotechnological discoveries have contributed to a deeper understanding of the role of these enzymes in cellular homeostasis and body physiology (de Góes-Favoni and Bueno 2014; Martins and Choupina 2018). Among the transglutaminases, blood coagulation factor XIII is involved in cross-linking between fibrin molecules (Ramanujam et al. 2025). Its function results in the formation of fibrin clots. However, this type of enzyme is rarely used in the food industry because it transfers red color to food and requires thrombin activation. Furthermore, it can lead to protein precipitation in products containing, for example, casein (Zhang et al. 2009; de Góes-Favoni and Bueno 2014; Kieliszek and Błażejak 2017). A breakthrough in transglutaminase research occurred in the 1980 s thanks to scientists from Ajinomoto Co., Inc., a Japanese food and chemical company (Yamaguchi 2017). It is worth emphasizing that microbial transglutaminases, unlike their animal counterparts, can be produced extracellularly by various microbial strains (Nagy and Szakacs 2008; Yuan et al 2024a). These technological features have generated considerable scientific interest due to the stability and suitability of these enzymes for industrial processing of multiple products. Preliminary studies have shown that some strains of bacteria (*Actinobacteria*) possess the ability to biosynthesize transglutaminase (Kolotylo et al 2024b). Scientists have identified an enzyme derived from *Streptomyces mobaraensis*, which they isolated from a soil sample in Mobara, Japan (Tan et al. 2019; Miwa 2020). The development of this field marked the beginning of further, in-depth biotechnological research. Subsequent studies have confirmed that microbial transglutaminase (mTG) is produced primarily by soil actinomycetes (Martins et al 2014; Yuan et al 2024a). *Streptomyces* species represent the most metabolically efficient organisms capable of producing this enzyme commercially. Importantly, mTG demonstrates high catalytic efficiency toward food proteins and greater tolerance to processing conditions compared to animal TG (Yuan 2024a) . These processes have led to the increasingly widespread use of mTG in the food industry. Literature data (Deng et al. 2025; Mostafa 2020) confirm that mTG is widely used to restructure protein networks in various food matrices. This enzyme enhances texture, elasticity, and water retention capacity, while also improving the stability of emulsions and foams, and extending the shelf life of various products (Redd et al 2024). These properties justify its widespread use in meat, fish, dairy, bakery, and plant-based products (Zimoch-Korzycka et al 2024; Akbari et al 2021). It is worth noting that they were the first in the world to produce this enzyme on an industrial scale and successfully commercialize it in Japan under the brand name “*Activa*” in the early 1990 s (Liu et al. 2024a). Figure 1 provides a historical overview of transglutaminase research.

Unlike many other transglutaminases, calcium does not regulate microbial isoforms (Kolotylo et al. 2023), making them a more versatile and suitable enzyme form for applications in the food industry. Furthermore, unlike mammalian tissue transglutaminases such as TG2, whose activation requires the presence of Ca^2^⁺ ions, microbial transglutaminases do not possess functional Ca^2^-binding domains (Tagami et al. 2009). In mammalian enzymes, calcium ions induce a transition from the closed to the open conformation of the enzyme (the catalytically active form of the protein) (Savoca et al. 2018). The crystal structure of the mTG protein demonstrates that the active site (Cys–His–Asp catalytic triad) (Kashiwagi et al. 2002) is permanently accessible without the need for ion-induced conformational changes. mTG occurs naturally as an extracellular enzyme, where Ca^2+^ concentrations are variable and difficult to control. Therefore, the catalytic activity of mTG is constitutive and independent of ionic cofactors (Duarte et al. 2020). Proteins such as milk casein, soy globulins, and myosin are sensitive to Ca^2+^ and easily precipitate (Kolotylo et al. 2023; Yokoyama 2024). The advantage of mTG over previously discovered TG enzymes, in addition to its independence from calcium ions (de Góes-Favoni and Bueno 2014), stems from its reduced molecular weight; the fact that the enzyme produced by microorganisms requires fewer purification processes; its stability over a wide range of temperatures and pH; and the possibility of obtaining it in large quantities via solid-state fermentation. All these features have favored the industrial applications of mTG, reducing costs and increasing its commercial role in food. It is worth noting that the industrial use of this protein quickly gained interest in many other countries. As research in this area continued, the possibilities for its further implementation began to be explored. It was primarily used to improve the texture, elasticity, and water-binding capacity of food proteins, as well as to increase the stability of emulsions and foams, and to extend the shelf life of food products in the meat, fish, dairy, bakery, and vegetarian/vegan industries (Duarte et al. 2020; Romeih et al. 2024; Yokoyama 2024).

## Characteristics of *Streptomyces*

**Fig. 2:**
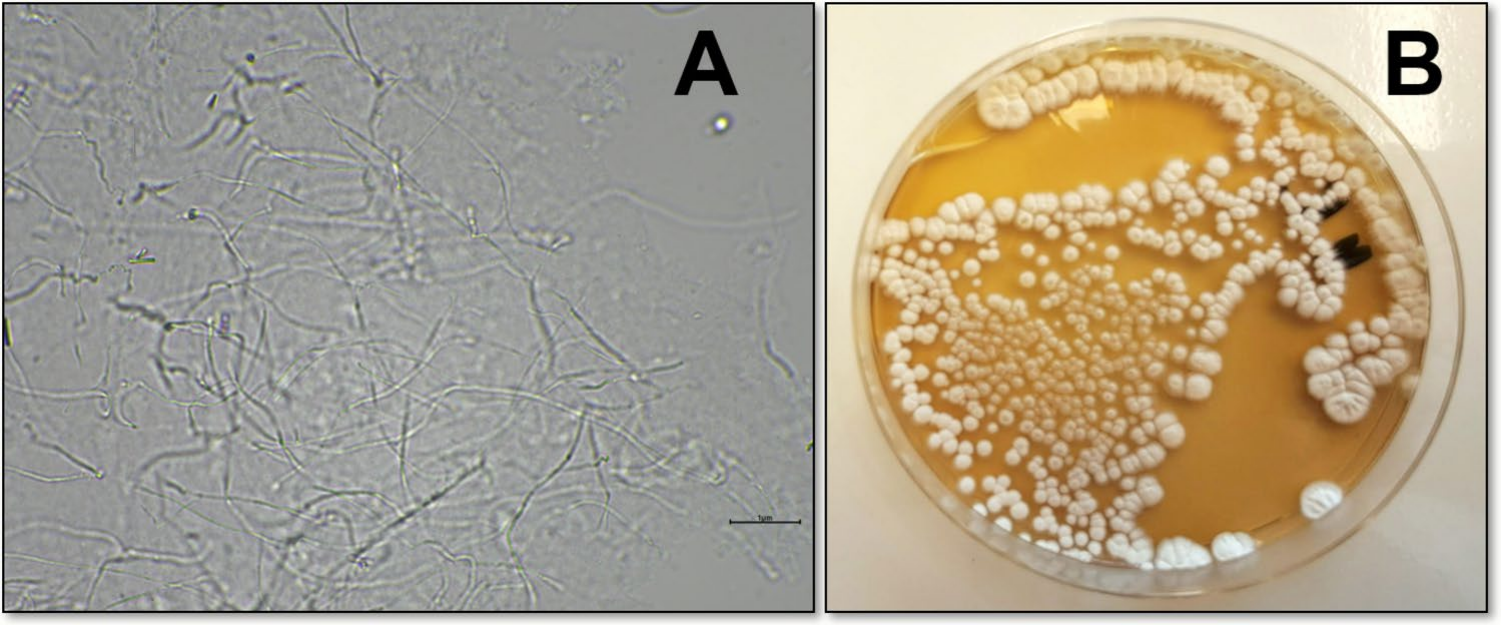
Characteristic macroscopic image (**A**) and morphological features (**B**) of *Streptoverticillium cinnamomeum* strain

*Streptomyces* is a Gram-positive bacterium from the *Streptomycetaceae* family and the *Actinobacteria phylum* (Hungund et al. 2021). These bacteria comprise 1340 species (https://lpsn.dsmz.de/search?word=streptomyces). These microorganisms are multicellular aerobes that form filamentous morphological structures (Tariq et al. 2025). Actinomycetes occur in soil, oceans, terrestrial plants, and various unexploited extreme environments (Barka et al. 2016; Law et al. 2018; Djebbah et al. 2021). Most species of these bacteria are mesophilic, with optimal growth temperatures ranging from 25 to 37 °C. Some *Streptomyces* species can adapt to higher temperatures, reaching 60 °C (Chouyia et al. 2022). Therefore, these microorganisms also occur in thermal spring areas (Duan et al. 2014; Amin et al. 2017). They are characterized by a high guanine-cytosine (GC) content (over 55%) in their DNA (Lertcanawanichakul and Sahabuddeen 2023; Le et al. 2024). *Streptomyces* have special features such as a complex life cycle involving mycelial development (Jones and Elliot 2018; Zambri et al. 2022) with extensive branching containing LL-diaminopimelic acid as the major diamino acid (Lee et al. 2023) and aerial hyphae capable of differentiating into spores or arthrospores (Fig. 2A) (Butt et al. 2024). The complex life cycle of *Streptomyces*, which includes mycelial differentiation and pellet formation, is a key factor in determining the efficiency of enzyme biosynthesis (Manteca Fernández and Yagüe Menéndez 2018). Furthermore, optimal microbial biomass production and the presence of various determinants, such as oxygen, are essential for industrial transglutaminase production (Barbuto Ferraiuolo et al. 2021). The regulatory and metabolic technological properties of these strains also play a key role (Xu et al. 2022). They are increasingly being used in studies to enhance mTG biosynthesis using classical metabolic engineering methods (Xu et al. 2022). It is worth noting that *Streptomyces* can synthesize several pigments responsible for the aerial mycelium's coloration (Fig. 2B) (Bhat and Nayaka 2023; Ramya et al. 2025). It is also worth emphasizing that *Streptomyces* is a key element in soil ecosystems, playing a role in the decomposition of organic matter (Al-Quwaie 2023), e.g., wood components (Thakur et al. 2025). Furthermore, they play a role in plant protection, helping to fight plant pathogens (Pacios-Michelena et al. 2021; Al-Quwaie 2023). Therefore, they are essential not only from an ecological perspective, but also from an agricultural and environmental perspective. Importantly, some strains of actinomycetes can exist in symbiosis with marine organisms (Chen et al. 2021), as well as plant root symbionts in the rhizosphere (Nicolle et al. 2024; Liu et al. 2024b).

It is worth emphasizing that in submerged fermentation (SMF), the formation of branched hyphae by *Streptomyces* often leads to increased viscosity (van Dissel et al. 2014) and heterogeneous access to oxygen (Zacchetti et al 2016) and nutrients. On the other hand, the production of compact mycelial aggregates in SMF can improve oxygen diffusion into the internal biomass and increase the stability of TG production (van Dissel et al. 2014). The controlled aggregate size of this strain has been shown to correlate positively with productivity and repeatability in industrial fermentations. In solid-state fermentation (SSF), the hyphal form of *Streptomyces* is particularly advantageous. The ability to colonize solid substrates through extensive hyphal penetration enables the effective utilization of agricultural and industrial residues (Yegin 2025). SSF systems better reflect the natural habitat of *Streptomyces*, which is crucial for the biosynthesis of secondary metabolites (Kumar et al 2021). Furthermore, such morphological features can offer technological advantages.

*Streptomyces* has long been a fascinating group of microorganisms, continually studied for its production of biologically active secondary compounds (Lacey and Rutledge 2022). According to Anderson and Wellington (2001), over 10,000 bioactive compounds have been documented. Lacey and Rutledge (2022) believe that *Streptomyces* can produce up to 150,000 metabolites. Notably, their metabolites (including antibiotics, anticancer agents, immunosuppressants, and pigments) are widely used in various industries. The discovery of streptomycin (derived from *Streptomyces griseus*) (Alekseeva et al. 2023) and actinomycin (derived from *Streptomyces antibioticus*) (Machushynets et al. 2022) revolutionized antibacterial and oncological therapy. Approximately 75% of known antibiotics are isolated from actinomycetes, and two-thirds are produced from *Streptomyces* (Lertcanawanichakul and Sahabuddeen 2023). Furthermore, the ongoing development of this field of science leads to the continuous exploration of this group of microorganisms in search of new, potential bioactive substances with therapeutic and industrial properties. Streptomycin, the first widely used antibiotic against tuberculosis (Wei et al. 2022; Patel et al. 2025), and actinomycin, which exhibits potent anticancer activity (Aftab and Sajid 2017), represent only a fraction of the enormous potential of this group of microorganisms. In addition to producing antibiotics, *Streptomyces* produces enzymes necessary in food biotechnology. One such enzyme is transglutaminase (Fuchsbauer 2022), which catalyzes the formation of bonds between glutamine and lysine molecules in proteins (Kang et al. 2024). Such properties lead to structural modification of proteins in various food products and improvement of their physical properties (Mattice and Marangoni 2021). The production of transglutaminase by *Streptomyces* bacteria creates new opportunities for multiple industries. Thanks to such properties, creating products of higher quality and better sensory and nutritional properties is possible.

## Characteristics of transglutaminase and its mechanism of action

**Fig. 3:**
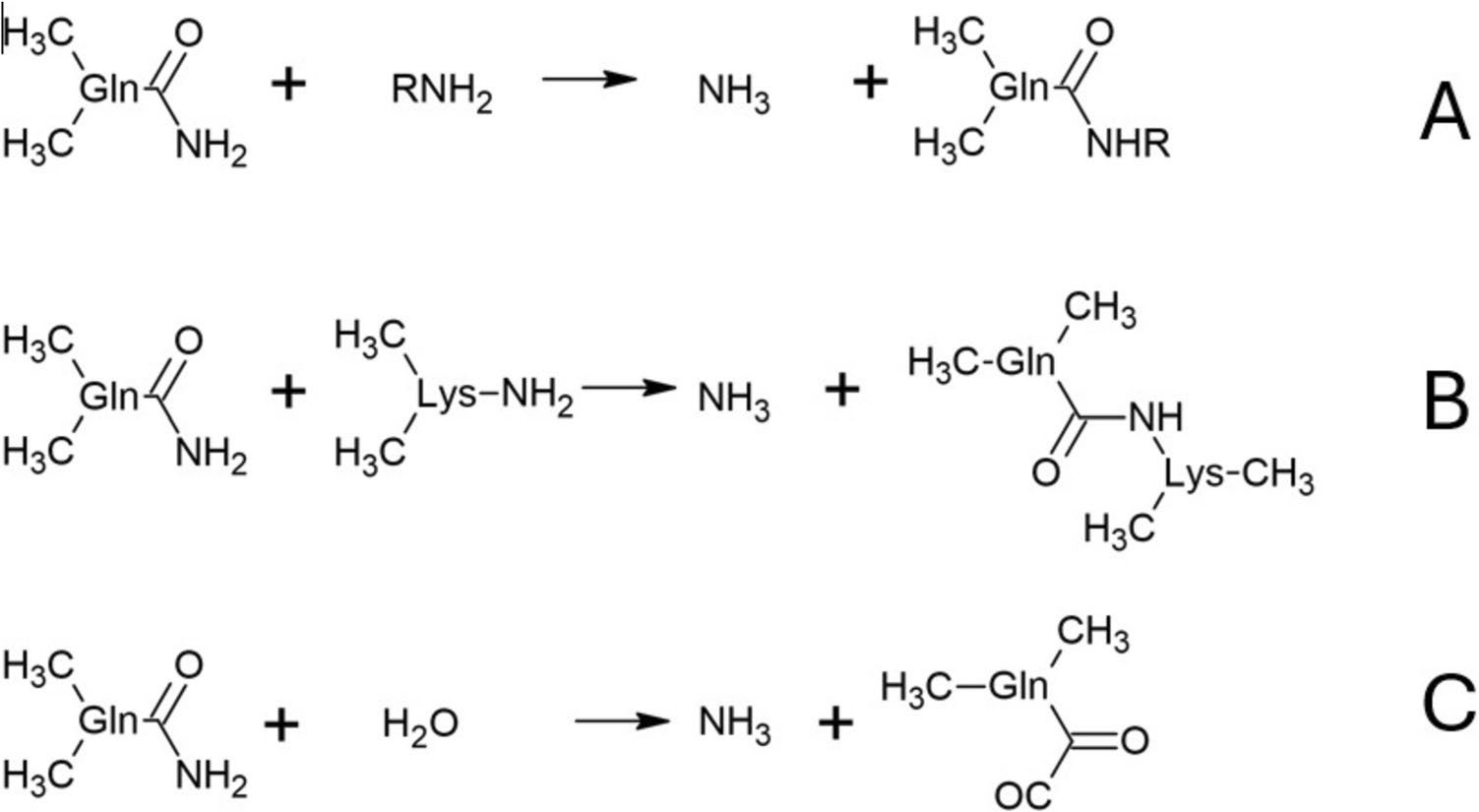
Reactions catalyzed by transglutaminase. **A** The formation of a covalent amide bond between the γ-carboxamide group of a glutamine residue in one protein and the ε-amino group of a lysine residue in another protein, leading to the formation of isopeptide cross-links between the proteins. **B** In a transamidation reaction, a primary amine attaches to a glutamine residue in the protein. **C** A water molecule can act as a nucleophile, leading to a deamidation reaction: the conversion of glutamine to glutamic acid (glutamate)

Transglutaminases are a group of enzymes belonging to the transferase class (EC 2), which includes enzymes that catalyze the transfer of functional groups between different molecules (Chen et al. 2023; Wang et al. 2023). Within this class, transglutaminases are classified in the EC 2.3.2. subclass, aminoacyltransferases, enzymes responsible for forming covalent bonds (Fischer 2024). Microbial transglutaminase (mTG) can catalyze the formation of cross-links between protein molecules, which is widely used in modifying the functional properties of food. This enzyme belongs to the transglutaminase family, including human tissue transglutaminase (tTG) (Matthias et al. 2016). Microbial transglutaminase catalyzes an acyl transfer reaction in which the γ-carboxamide group of the glutamine residue forms a thioester intermediate with cysteine in the enzyme’s active site (Fig. 3) (Kieliszek and Misiewicz 2014). The ε-amino group of the lysine residue then acts as an acyl acceptor, leading to the formation of a stable γ-glutamyl-ε-lysine isopeptide bond (Miwa 2020; Velazquez-Dominguez et al. 2023). In the absence of suitable amine donors, water can act as a nucleophile, leading to deamidation rather than cross-linking (Vasić et al 2023). The exceptional stability of the γ-glutamyl-ε-lysine bond under conditions of high temperature, proteolysis, and pH changes underlies the technological characteristics of this enzyme in food processing, particularly in the restructuring of protein matrices, e.g., meat and dairy products (Vasić et al 2023). It is worth noting that reactions catalyzed by transglutaminases enable modification of the protein network structure, leading to enhanced properties such as gel formation and water retention (Romeih and Walker 2017). These properties are widely used in the food industry to improve the texture and functionality of various food products.

Depending on their source, transglutaminases are divided into animal, plant, and microbial (Kolotylo et al. 2023) (Table 1). They can occur in oligomeric forms, such as monomers, dimers, or tetramers (Vasić et al. 2023). Furthermore, they differ in structure, specificity of action, and biochemical properties (Del Duca and Cai 2024). It is worth noting that human type 2 transglutaminase consists of four domains: an N-terminal β-sandwich, a catalytic domain containing the Cys-His-Asp catalytic triad, and two C-terminal β-barrels (Strop 2014). Human and animal transglutaminases play a key role in processes such as cell adhesion, blood coagulation, wound healing, and extracellular matrix stabilization (Poole et al. 2022; Liu et al. 2024a; Yongsawatdigul et al. 2024). It is worth emphasizing that microbial transglutaminase differs from human tissue transglutaminase 2 (TG2) in terms of structure, regulatory activity, and physiological function. Microbial transglutaminase lacks the regulatory domains characteristic of mammalian transglutaminases. Furthermore, TG2 contains several functional domains that enable it to interact with membrane receptors, integrins, and extracellular matrix components (Sewa et al. 2024). It is worth noting that TG2 plays a key role in the pathogenesis of celiac disease (Klöck et al. 2012), where it catalyzes the deamidation of gluten peptides (Stricker et al. 2022). The consequence of these processes is an increased affinity for HLA-DQ2/DQ8 molecules, resulting in an enhanced autoimmune response (Tjon et al. 2010; Wei et al. 2020). Microbial transglutaminase is not an endogenous enzyme and does not participate in antigen presentation processes (Stricker et al. 2022). Literature data do not support the possibility that mTG can initiate an autoimmune response analogous to that in which TG2 participates (Lerner et al 2023) have evaluated microbial transglutaminase as a food processing aid (Zorn et al 2024). Furthermore, the organizations have demonstrated that under approved conditions of use, this enzyme does not pose a safety risk. Current evidence does not support a direct immunogenic effect of mTG itself, but research is ongoing into its indirect role in modifying protein substrates.

**Table 1:** Comparative characteristics of selected types of transglutaminase enzymes

Features	Microbial transglutaminase (mTG)	Plant transglutaminase (pTG)	Animal transglutaminase
Source and occurrence	Bacteria, e.g., *Streptomyces mobaraensis*	Leaves, seeds, chloroplasts, heterogeneous proteins with TG properties (Parrotta et al 2022)	Mammals (multiple tissue isoenzymes), plasma: Factor XIII (FXIIIa) (Eckert et al 2014)
Molecular weight	Typically smaller, approx. 30–60 kDa	Varied mass, various isoforms occur (Parrotta et al 2022)	Large, multidomain proteins (e.g., TG2, approximately 70 kDa). FXIII has subunits (A2B2) (Kim and Park 2020)
Ca^2^⁺ ion dependence	Ca^2^⁺-independent (Akbari et al 2021; Savoca et al 2018)	Ca^2^⁺-dependent (Parrotta et al 2022)	Ca^2^⁺-dependent (Eckert et al 2014)
Substrate specificity	Very large (Kieliszek and Misiewicz 2014)	Substrates related to plant functions (Parrotta et al 2022)	High specificity, TG2 targets various substrates in the extracellular and intracellular matrix (e.g., fibrin, VWF, cytoskeletal proteins) (Eckert et al 2014; Rauhavirta et al 2016)
Catalytic mechanism	Similar mechanism (conventionally cysteine nucleophile), but different domain architecture and active environment; lack of Ca^2^⁺ requirement influences conformation (Savoca et al 2018)	Cysteine residue; regulation by Ca^2^⁺ alters access to the active site (Parrotta et al 2022)	Mechanism with a Cys-His-Asp residue in the active site (Kim and Park 2020)
pH, temperature optima, and stability	Stability over a wide range of pH and temperatures (dependent on the specific protein), easier storage and use in food (Kieliszek and Misiewicz 2014; Akbari et al 2021)	Various, often adapted to plant cellular conditions (Parrotta et al 2022)	Variable; somatic enzymes regulated by the cellular environment, TG2 shows susceptibility to redox regulation (Kim and Park 2020)
Activity regulation (modulating factors)	Activity depends on the availability of substrates and environmental conditions. It can be inhibited or activated by specific inhibitors (Savoca et al 2018; Kieliszek and Misiewicz 2014)	Activity modulated by Ca^2^⁺, phosphorylation, interactions with plant proteins, and stress signals (Parrotta et al 2022)	Strong regulation: Ca^2^⁺, redox, nucleotides (GTP/ATP), phosphorylations, and interactions with the extracellular matrix (ECM) (Eckert et al 2014)

Microbial forms of TG are used, among others, in the food industry to modify protein properties (Lerner et al. 2025; Wang et al. 2025). Plant transglutaminases, in turn, are characterized by a unique profile of catalytic activity (Serafini-Fracassini et al. 2009). The first evidence of the presence of transglutaminases in plants was reported in 1987 (Parrotta et al. 2022). Plant TG is essential in photosynthesis, plant growth, and development (Martins et al. 2014; Parrotta et al. 2022; Brus-Szkalej et al. 2025). Its various isoforms are known to be located in organs and parts of plant cells, including leaves, tubers, roots, flowers, buds, pollen, and different cellular compartments, including chloroplasts, the cytoplasm, and the cell wall (Aloisi et al. 2016). It is worth noting that plant transglutaminases are also believed to participate in processes related to the formation of cell structures and in plant defense responses to stress factors (Serafini-Fracassini et al. 2021; Del Duca and Cai 2024). As reported by Zou et al. (2024), transglutaminases of plant origin exhibit low activity, poor stability, and complex purification processes, which seriously limit the industrial use of this plant enzyme. Transglutaminases of animal and plant origin, although they do not show homology in the amino acid sequence, are characterized by similar catalytic activity and biochemical properties to microbial transglutaminases (Kieliszek and Misiewicz 2014; Vasić et al. 2023).

It is worth emphasizing that in recent years, there has been a noticeable increase in interest in microbial transglutaminase as a potential substitute for traditional enzymes of plant and animal origin. These changes stem from the search for more effective and economical solutions in the food industry and growing consumer awareness. The enzymatic properties of microbial transglutaminase, obtained primarily from bacteria and fungi, have effectively replaced the expensive and controversial animal enzyme derived from guinea pigs. Such efforts have led to dramatic progress in food production using transglutaminase. Microbial transglutaminase demonstrates broad stability under various process conditions (Lauber et al. 2001; Menéndez et al. 2006; Cui et al. 2008). For this reason, it is the preferred solution used in various areas of the food and biotechnology industries (Kieliszek and Misiewicz 2014; Brus-Szkalej et al. 2025). Microbial transglutaminases are attractive enzymes for industrial applications, especially in the functional food sector. This is primarily due to the need to develop alternative enzyme sources acceptable to consumers. Importantly, the continuous development of biotechnological methods has enabled genetic engineering to modify microorganisms to increase transglutaminase production (Yin et al. 2021; Yokoyama 2024; Aqeel et al. 2024). Such activities optimize the activity and stability of produced enzymes under various industrial conditions. Studies on the crystal structure of transglutaminases have also provided important information on the mechanism of their action and the possibility of modifying the enzyme at the molecular level (Kashiwagi et al. 2002; Song et al. 2022).

Microbial transglutaminase from *Streptomyces* is secreted as a proenzyme that can be activated by several exogenous proteases (bovine trypsin, intestinal chymotrypsin, or *Bacillus polymyxa* dispase) (Chen et al. 2013). Microbial transglutaminase is composed of 331 amino acid residues (Bauer et al. 2025) occurring on a single polypeptide chain. The secondary structure of this enzyme consists of eight β-strands surrounded by eleven α-helices (Vasić et al. 2023). It is worth noting that a metalloprotease was isolated from *Streptomyces mobaraensis* as an endogenous protease activating transglutaminase (Fuchsbauer 2022). Furthermore, it has been shown that not only an endogenous metalloprotease but also an endogenous serine protease is involved in the transglutaminase activation process by *Streptomyces hygroscopicus* (Fuchsbauer 2022; Ye et al. 2024). In *Streptomyces* strains, pro-MTG exhibits a conserved amino acid sequence (Zhang et al. 2009). They contain protease recognition sites located upstream of the N-terminus of the enzyme chain. The activating protease (TAMEP) catalyzes the cleavage of 41 amino acids at the N-terminus of the microbial transglutaminase (pro-mTG) zymogen, resulting in the formation of the active enzyme (Shi et al. 2024). Furthermore, enzyme activation can be blocked by a protease inhibitor, which is responsible for its activation (Zhang et al. 2009). N-terminal amino acid sequencing and homology analysis of the purified transglutaminase-activating protease inhibitor revealed that it is a member of the *Streptomyces* subtilisin inhibitor family (Fuchsbauer 2022). It is worth noting that transglutaminase is secreted and activated during cell differentiation, not during cell growth (Zhang et al. 2009). Furthermore, *S. hygroscopicus* differentiation and transglutaminase are inhibited by cystamine (Fuchsbauer 2022; Luan et al. 2022).

In summary, it is worth noting that transglutaminases perform different functions depending on their origin. However, it is important to emphasize that their primary role is the structural modification of proteins. This process is crucial for maintaining the proper functioning of organisms and their adaptability in response to changing environmental conditions.

## Basics of transglutaminase production

In 1987, Ajinomoto Co., Inc., while screening soil samples, discovered a strain of the bacterium *Streptomyces mobaraensis*. This microorganism was found to produce the highly active extracellular enzyme transglutaminase (TG) (Miwa 2020). The resulting enzyme had structural properties different from those of previously known TG extracted from mammalian tissues. Transglutaminase from *S. mobaraensis* is a 37.9 kDa monomer with an isoelectric point 8.9 (Shen et al. 2022). The enzyme is active at pH 4 and higher (Lerner and Benzvi 2021) with an optimal pH range of 6–7 (Hebishy et al. 2022; Kolotylo et al. 2024a). It is worth emphasizing that its mass is significantly smaller than that of mammalian TGs (Sadowska and Diowksz 2016). Furthermore, this protein is characterized by a typically spherical conformation of eight β-sheets surrounded by eleven α-helices. The enzyme’s structure resembles a disc with a clearly marked lateral cleft. Within this cleft, a cysteine residue is located at position 64. This cleft contains a free thiol group, which determines the protein’s enzymatic activity (de Góes-Favoni and Bueno 2014; Kieliszek and Misiewicz 2014; Lerner and Matthias 2020). Today, microbial transglutaminase is used industrially due to its lower cost and more straightforward production process. Furthermore, the distinct properties of microbial enzymes play a crucial role. mTG is characterized by a higher reaction rate, thermostability, Ca^2+^ independence (Ono et al. 2025), lower deamidation activity (Heil et al. 2016), oraz szerszą specyficznością substratową (Duarte et al. 2020). Since 1998, this enzyme has been approved by the FDA (Food and Drug Administration) as GRAS (Generally Recognized as Safe), i.e., safe for human consumption (Wang et al. 2018).

The first method for obtaining transglutaminase was extracting and purifying the enzyme from animal body fluids and tissues. Commercial production of this enzyme began in the 1970 s (Duarte et al. 2021) from the blood of slaughter cattle (Yokoyama 2024). This enzyme was initially used in medicine to prevent and treat bleeding (Dickneite et al. 2015). However, its production from animal tissue was unprofitable due to the costs associated with obtaining and purifying transglutaminase (factor XIII). With the development of biotechnology, more efficient methods for producing transglutaminase using microorganisms emerged, which proved to be more effective and safe (Kolotylo et al. 2023). These aspects allowed for increased enzyme production more sustainably and economically.

Another method for obtaining transglutaminase involves the controlled submerged fermentation (SMF) of appropriately selected microbial strains. Fermentation is a technique for biologically converting complex substrates into simple compounds by various microorganisms. This process enables efficient enzyme biosynthesis under industrial conditions, maintaining high yields and catalytic activity of the final product. Submerged fermentation (SMF) involves inoculating a microbial culture into a liquid medium to produce the desired product (Singhania et al. 2015). Furthermore, the substrate degradation process by the microorganisms occurs in large amounts of water (Melini and Melini 2024) and using a highly oxygenated growth medium (Shabeer and Mishra 2025). Some typical substrates used in submerged fermentation include soluble sugars, molasses, and fruit and vegetable juices. Submerged fermentation is more commonly used for enzyme production than solid-state fermentation mainly due to the ability to control parameters using appropriate substrates (Krishna 2005).

Another possibility for obtaining transglutaminase is solid-state fermentation (SSF) (Ja’afar and Shitu 2022). The process uses a solid, insoluble substrate with limited access to water (De Villa et al. 2023). A significant advantage of this fermentation is the ability to use waste from the agri-food industry as components of the growth medium for microorganisms (Bibi et al. 2023). Such activities contribute to the effective management of agricultural waste, thus reducing the negative impact of the agri-food industry on the environment (Khan et al. 2024; Yadav et al. 2024). The use of natural waste, which is rich in carbohydrates, proteins, and other nutrients, offers the possibility of increasing the profitability of the process while simultaneously obtaining transglutaminase (Ravindran et al. 2018; Vasić et al. 2023). In most cases, wheat bran is used for the production of extracellular enzymes (Fatima et al. 2019), but transglutaminase production from fisheries and aquaculture processing (Khiari 2024), rapeseed meal, wheat straw, cottonseed, or rice bran has also been described (Fatima et al. 2019). Solid-state fermentation is characterized by high yields of obtained products (Krishna 2005; Thomas et al. 2013).

The most commonly used microorganisms in transglutaminase production are *Streptomyces mobaraensis*, *Streptoverticillium cinnamoneum*, *Actinomadura* sp., *Streptoverticillium ladakanum*, *Bacillus circulans*, and *Streptomyces hygroscopicus* (Duarte et al. 2020)*.* A comparison of transglutaminase production by selected strains is summarized in Table 2. It is worth noting that environmental factors play a key role in the mTG biosynthesis process. The most important parameters include pH, fermentation temperature, and the type and availability of carbon and nitrogen sources. Numerous approaches to optimizing these variables to maximize enzymatic production have been described in the literature (Fatima and Khare 2021; Aqeel et al. 2024; Chang et al. 2025). Despite its limitations, one of the most commonly used optimization methods is the “*One factor at a time (OFAT)*” strategy. It involves modifying a single experimental parameter, such as temperature or pH, while maintaining other factors constant. Although this method is time-consuming and does not account for interactions between factors, it remains a useful tool in preliminary optimization studies (Macedo et al. 2007; Bahrim et al. 2010). Another method that is increasingly being used in optimizing enzyme production is Response Surface Methodology (RSM). This method enables the simultaneous analysis of the effects of multiple factors and their interactions. Furthermore, RSM allows for more efficient and precise determination of optimal process conditions (Ghosh and Lonhare 2024) contributing to increased transglutaminase biosynthesis efficiency (Souza et al. 2006; Kolotylo et al. 2024b). The following sections discuss strategies for biotechnological transglutaminase production.

**Table 2:** Natural microbial sources of transglutaminase

Microorganisms	Medium	Fermentation scale	Incubation time	Transglutaminase activity	References
*Bacillus subtilis* isolate B4	Peptone 2%, starch 2%, yeast extract 0.2%, Mg_2_SO_4_·7H_2_0 0.2%, K_2_HPO_4_ 0.2%, KH_2_PO_4_ 0.2%, pH 7.0	Flasks	48–60 h	3.95 U/mL	Sorde and Ananthanarayan (2019)
*Enterobacter* sp. C2361	Soluble starch 2%, peptone 2%, yeast extract 0.2%, Mg_2_SO_4_ 0.2%, K_2_HPO_4_ 0.2%, KH_2_PO_4_ 0.2%, pH 7.0	5-mL tube	48 h	0.87 U/mL	Bourneow et al. (2012)
*Providencia* sp. C1112	Soluble starch 2%, peptone 2%, yeast extract 0.2%, Mg_2_SO_4_ 0.2%, K_2_HPO_4_ 0.2%, KH_2_PO_4_ 0.2%, pH 7.0	5-mL tube	42 h	0.92 U/mL	Bourneow et al. (2012)
*Streptomyces mobaraensis* NCIM 5208	Wheat bran 5 g suspended in 20 mL minimal salts solution consisting: g/L of KH_2_PO_4_ 5, NH_4_NO_3_ 1, MgSO_4_·7H_2_O 1, NaCl 1, CoCl_2_·6H_2_O 0.001, MnSO_4_ 0.0008, ZnSO_4_·7H_2_O 0.0017; FeSO_4_·7H_2_0 0.0025, pH 6.0	500-mL flasks	15 days	3.318 U/mL	Fatima et al. (2019)
*Streptoverticillium mobaraensis* S-SI12	Polypepton 2.0% glucose 2.0% K_2_HPO_4_ 0.2%, MgSO_4_ 0.1%, yeast extract 0.2%, pH 7.0	500-mL flasks	48 h	2.5 U/mL	Ando et al. (1989)
*Streptomyces* sp. CBMAI 1617 (B6 strain)	Soybean meal 2.5%, potato starch 2%, glucose 0.1%, bacteriological peptone 0.1%, KH_2_PO4·7H_2_O 0.4%, MgSO_4_·7H_2_O 0.2%, pH 7.0	500-mL flasks	96 h	6.07 U/mL	Ceresino et al. (2018)
*Streptoverticillium ladakanum* NRRL-3191	Xylose 2%, yeast extract 0.25%, peptone 0.10 g/L, MgSO_4_ 0.05%, KH_2_PO_4_ 0.2%, Na_2_HPO_4_ 0.5%, C_20_H_27_FN_2_ 0.2%	50-mL flasks	72 h	0.348 U/mL	Téllez Luis et al. 2004)
*Actinomadura* sp. T-2	Polypeptone 2%, soluble starch 2%, yeast extract 0.2%, K_2_HPO_4_ 0.2%, 0.1% MgSO_4_·7H_2_O, pH 7.0	500-mL flasks	120 h	2.26 U/mL	Kim et al. (2000)
*Actinomadura* sp. T-2	Glucose 2%, polypeptone 2%, soluble starch 2%, yeast extract 0.2%, K_2_HPO_4_ 0.2%, 0.1% MgSO_4_·7H_2_O, pH 7.0	500-mL flasks	120 h	5.05 U/mL	Kim et al. (2000)
*Streptoverticillium cinnamoneum* KKP 1658	Soluble starch 2%, peptone-aminobak 2%, yeast extract 0.2%, KH_2_PO_4_ 0.2%, Na_2_HPO_4_ 0.2%, MgSO_4_·7H_2_O 0.1%, pH 6.0–6.5	500-mL flasks	72 h	6.59 U/mL	Kolotylo et al. (2024a)
*Streptoverticillium cinnamoneum* KKP 1658	Soluble starch 2%, peptone-aminobak and corn steep liquor: 2.75% nitrogen, yeast extract 0.2%, KH_2_PO_4_ 0.2%, Na_2_HPO_4_·12H_2_O 0.2%, MgSO_4_·7H_2_O 0.1%, pH 6.5	Bioreactor	72 h	4.29 U/mL	Kolotylo et al. (2025)
*Streptomyces paucisporogenes* ATCC 12596	Liver kidney bean 67%, KH_2_PO_4_ 0.5%, NH_4_NO_3_ 0.5%; MgSO_4_·7H_2_O 0.1%, NaCl 0.1%, CoCl_2_·6H_2_O 1 mg/L, MnSO_4_ 0.8 mg/L, ZnSO_4_·7H_2_O 1.7 mg/L, FeSO_4_·7H_2_O 2.5 mg/L, pH 6.0	500-mL flasks	96 h	4.2 U/g	Nagy and Szakacs (2008)
*Streptomyces platensis* NRRL 2364	Liver kidney bean 67%, KH_2_PO_4_ 0.5%, NH_4_NO_3_ 0.5%; MgSO_4_·7H_2_O 0.1%, NaCl 0.1%, CoCl_2_·6H_2_O 1 mg/L, MnSO_4_ 0.8 mg/L, ZnSO_4_·7H_2_O 1.7 mg/L, FeSO_4_·7H_2_O 2.5 mg/L, pH 6.0	500-mL flasks	7 days	5.1 U/g	Nagy and Szakacs (2008)
*Streptomyces fradiae* ATCC10745	Soluble starch 1%, KH_2_PO_4_ 0.1%, NaCl 0.1%, MgSO_4_·7H_2_O 0.1%, CaCO_3_ 0.2%, (NH_4_)_2_SO_4_ 0.2%, MnSO_4_·7H_2_O 0.0001%, ZnSO_4_·7H_2_O 0.0001%, FeSO_4_·7H_2_O 0.0001%, glycerol 1%, casein 1%, peptone 1%, yeast extract 1%, pH 7.2	-	168 h	0.29 U/mL	Merajian and Asoodeh (2025)
*Streptoverticillium cinnamoneum* CBS 683.68	Soya peptones 0.1%, glicerol 0.4%, MgSO_4_ 0.05%, KH_2_PO_4_ 0.2%, Na_2_HPO_4_ 0.5%, yeast extract 0.4%, oligoelements: FeSO_4_·7H_2_O, ZnSO_4_·7H_2_O, MnSO_4_·7H_2_O 0.0001% each	Flasks	120 h	0.1 U/mL	Junqua et al. (1997)
*Streptoverticillium cinnamoneum* CBS 683.68	Casein 3.84%, peptone 0.105%, yeast extract 0.25%, MgSO_4_ 0.05%, KH_2_PO_4_ 0.2%, Na_2_HPO_4_ 0.5%	Flasks	Approximately after 200 h	0.331 U/mL	Junqua et al. (1997)
*Bacillus amyloliquefaciens* XCT-09	Pepton 1%, yeast extract 0.5%, NaCl 1%	Flasks with 25 mL of fermentation medium	48 h	0.373 U/mL	Zou et al. (2024)
*Streptomyces platensis* DSM 40041	Yeast extract 0.7%, glucose 1%, polypeptone 0.7%, soluble starch 3%, NaCl 3%, CaCO_3_ 0.5%, pH 7.0	Flasks	96 h	2.4 U/mL	Bech et al. (2000)
*Streptomyces lydicus* NRRL B-3446 and DSM 40555	Yeast extract 2%, glucose 1.2%, bactopeptone 0.001%, pluronic 0.001%, pH 6.5	Flasks	96 h	3.0 U/mL	Bech et al. (2000)
*Cercospora carisis* IMI 167.425	Potato meal 7.5%, BAN 800 mg 0.0075%, soy meal 4%, Na_2_HPO_4_ 0.9%, KH_2_PO_4_ 0.15%, 0.1 ml/L pluronic	Flasks	3–30 days	13 U/mL	Bech et al. (2000)

### Microbiological acquisition of transglutaminase

Microbiologically obtained transglutaminase is one of the most important achievements of modern industrial biotechnology. Microbial sources of transglutaminase enable the production of this enzyme, characterized by favorable technological properties and high efficiency under industrial process conditions.

In recent years, there has been growing interest in using various agricultural wastes as components of microbiological media. Due to their availability, low price, and potential for sustainable waste utilization in industrial biotechnology for producing valuable bioproducts, such as enzymes, these raw materials are the subject of numerous studies (Ravindran et al. 2018). Table 2 presents examples of mTG biosynthesis by selected strains of microorganisms. Research conducted by Fatima et al. (2019) demonstrated that TG can be obtained by using agricultural wastes as culture medium components. Strain *Streptomyces mobaraensis* NCIM 5208 was grown at 30 °C and pH 6.0 on media containing, among others, wheat bran, rice bran, soybean meal, potato hydrolysate, wheat straw, *k*-carrageenan, cottonseed, and castor bean seeds. The most effective mTG production (12.949 U/g) was achieved using wheat bran. de Souza et al. (2008) used industrial waste based on fibrous soybean residues to culture the *Bacillus circulans* BL32 strain for transglutaminase biosynthesis. The highest specific mTG activity was 0.249 U/mg protein. It is worth emphasizing that this raw material is characterized by a high protein and hemicellulose content, making it a particularly favorable component for this fermentation process. Glodowsky et al. (2020) cultivated an Arctic strain of *Penicillium chrysogenum* on a medium consisting of soybean hulls, obtaining TG activity of 4.87 mU. Maximum mTG activity was observed at pH 8.0 and 30 °C. Notably, using a chromatographic column with Q-sepharose IEX substrate in enzyme purification allowed obtaining a specific activity of 7.81 mU/mg. Guerra-Rodríguez i Vázquez (2013) conducted studies to assess potato hydrolysates’ suitability as an organic source in mTG production by the *Streptomyces mobaraensis* CECT 3230 strain. The study results clearly indicated that potato hydrolysates with both yeast extract and sodium caseinate significantly affected the efficiency of mTG synthesis at a level of 1.128 U/mL. Furthermore, potato hydrolysate was found to be a valuable and practical source of nutrients suitable for use in the production of this enzyme. According to Ceresino et al. (2018), the preparation of the medium used for TG biosynthesis by the *Streptomyces* sp. CBMAI 1617 (B6) strain showed that adding 0.5% glucose, 2.45% casein peptone, and 0.8% KH_2_PO_4_·7H_2_O had a key and significant effect on enzymatic activity. The highest TG activity was 6.074 U/mL. The study conducted by Portilla-Rivera et al. (2009) evaluated the effectiveness of using sugarcane molasses and glycerol as carbon sources in the TG biosynthesis process. *Streptomyces ladakanum* NRRL-3191 strain was used as the enzyme producer. The highest enzymatic activity (4.01 U/mL) was obtained using a 1:1 mixture of molasses and glycerol at pH 7.0 and 30 °C. These results indicate that both molasses and glycerol are suitable raw materials for the formulation of fermentation media, and their synergistic combination significantly increases the efficiency of microbial transglutaminase production. In the study by Sorde and Ananthanarayan (2019), it was found that the medium containing starch and peptone showed the highest efficiency in TG production. The highest enzymatic activity (3.95 U/mL) was recorded for isolate B4, which shows the most significant homology with the *Bacillus nakamurai* NRRL B-41091 strain. In turn, studies by Téllez-Luis et al. (2004) showed that the use of a medium containing 2% xylose in the strain culture was the most optimal condition for transglutaminase production by the *Streptoverticillium ladakanum* NRRL-3191 strain. The maximum enzymatic activity (0.348 U/mL) was obtained after 72 h of incubation. Nagy and Szakacs (2008) described the cultivation of *S. paucisporogenes* ATCC 12596 and *S. platensis* NRRL 2364 strains on solid media containing beans, peas, and lentils. The highest efficiency in transglutaminase production was demonstrated using kidney beans and mung beans as substrates. The optimal conditions for the biosynthesis of this enzyme included a temperature in the range of 45–50 °C and a pH of 5–9. Maximum TG activity for *Streptomyces paucisporogenes* and *Streptomyces platensis* strains reached 4.2 U/L and 5.1 U/L, respectively. Studies conducted by Kolotylo et al. (2024a, b) showed that the use of the genetically unmodified *Streptoverticillium cinnamoneum* strain KKP 1658 can be an effective method for mTG production. Tryptic soy broth (TSB) was selected as the optimal culture medium, ensuring high enzyme activity during incubation. The inoculum incubation time was 24 h with an inoculum dosage of 10%. Studies were also conducted with various nitrogen sources, maintaining a constant total nitrogen dose of 0.2% (including aminobac, corn liquor, ammonium nitrate, and ammonium sulfate) to optimize mTG production. The authors found that the highest mTG activity (6.50 U/mL) was obtained using a combination of aminobac with corn liquor for 72 h at 28 °C and pH 6.0–6.5, reaching 6.59 U/mL. Studies by Zheng et al. (2002) demonstrated that the highest transglutaminase activity was obtained when cultured at temperatures above 30 °C using the *Streptomyces mobaraense* WSH-Z2 strain. The maximum enzymatic activity was 2.94 U/mL. However, literature data (Böhme et al. 2020; Zou et al. 2024) show that the optimal catalytic temperature for mTG obtained after culturing the wild strain of *S. mobaraensis* was 50 °C, and its stability is poor when the temperature exceeds 60 °C. Thermal stability is an important indicator for evaluation in industrial applications, and TGs with higher thermal stability often have a wider range of applications. According to the data presented by Akbari et al. (2021), optimal conditions for enzyme production were found to include a temperature of approximately 30 °C and a pH of 7.0. The fermentation time should usually be from 72 to 96 h. Romeih and Walker (2017) argue that the optimal temperature for microbial transglutaminase is 40 to 50 °C. The optimal pH value for TG ranges from 5 to 7. The study presented by Xavier et al. (2017) demonstrated that TG obtained after cultivation by the strains *Streptomyces albereticuli* MTCC 323, *S. olivoverticilla* MTCC 333, *S. griseocarneus* MTCC 328, *S. septatus* MTCC 926, and *Bacillus* sp. MTCC 864 and MTCC 1434 showed the highest activity of this enzyme (0.46 U/mL) at pH 6.0 and 30 °C. Carbon and nitrogen sources are fundamental environmental aspects in transglutaminase production. Therefore, the following study by Macedo et al. (2007) conducted studies on selecting the most favorable nitrogen and carbon sources. The authors showed that the highest enzymatic activity (1.12 U/mL) was obtained using peptone as the nitrogen source. In the case of carbon sources, the best results were observed for a mixture of potato starch and glucose (1.12 U/mL) and maltodextrin (1.18 U/mL). It is worth emphasizing that the differences between these variants were not statistically significant. Bahrim et al. (2010) conducted studies on polar strains of *Streptomyces* sp. to evaluate their ability to biosynthesize transglutaminase under submerged fermentation conditions. The process was conducted at 28 °C and pH 6.0 for 10 days. Analysis of the composition of the fermentation media revealed that glucose, peptone, and potato starch significantly enhanced enzyme production. Among the isolates tested, the highest transglutaminase activity (approximately 0.8 U/mL) was observed for the *Streptomyces* sp. MIUG 13P strain, indicating its most significant biotechnological potential in industrial mTG production.

Microbiologically obtaining transglutaminase is a key direction in the development of enzymatic biotechnology. The presented production processes enable this enzyme’s controlled and sustainable production, which has a wide range of applications. The continuous development of biotechnological sciences and innovations in this field contributes to economic growth and enables the implementation of production technologies in many food industry sectors.

### Production of microbial transglutaminase using genetic engineering methods

Transglutaminase is typically obtained using microbial fermentation or recombinant DNA technology (Kieliszek and Misiewicz 2014; Vasić et al. 2023). Many research centers around the world are conducting research related to transglutaminase production in model microorganisms such as *Escherichia coli* and *Bacillus* (Duarte et al. 2021; Li et al. 2023; Zou et al. 2024)*.* Scientists have attempted to obtain high-efficiency transglutaminase expression using genetically modified strains. Table 3 presents examples of mTG biosynthesis by selected strains of microorganisms that have been genetically modified. Due to the well-known crystal structure of *Streptomyces* transglutaminase, the gene responsible for transglutaminase biosynthesis has become the best source of exogenous expression (Fuchsbauer 2022). Takehana et al. (1994) described a reconstructed transglutaminase-producing strain. The authors expressed the transglutaminase gene from *Streptoverticillium* S-8112 in *E. coli* JA221. The low activity of the produced enzyme (0.22 U/mg protein) makes this method unsuitable for commercial-scale transglutaminase production. However, it is worth emphasizing that genetic modifications can produce enzymes with improved properties. Such analyses allow for increased activity and improved production efficiency. Furthermore, this approach can lead to obtaining transglutaminase with appropriate enzymatic parameters.

**Table 3:** Examples of microorganisms genetically modified to produce transglutaminase

Microorganisms and expression system	Medium	Fermentation scale	Incubation time	Transglutaminase activity	Transformation method		References
*Streptomyces mobaraensis* smL2020 *Escherichia coli* ET12567/pUZ8002, plasmids pSET152 and pSET156	Glicerol 2%, peptone 2%, yeast extract 0.5%, corn steep liquor 0.5%, MgSO_4_·7H_2_O 0.2%, K_2_HPO_4_·3H_2_O 0.4%, KH_2_PO_4_·3H_2_O 0.2%, CaCO_3_ 0.2%, pH 7.4	250-mL flasks	48 h	56.43 U/mL	Signal peptide optimization, plasmid transformation		Yuan et al. (2024a)
		1000-L fermentor	42 h	63.18 U/mL			
*Streptomyces mobaraensis* S23Y/Y24N/S250R	Glicerol 2%, peptone 2%, yeast extract 0.5%, corn steep liquor 0.5%, MgSO_4_·7H_2_O 0.2%, K_2_HPO_4_·3H_2_O 0.4%, KH_2_PO_4_·3H_2_O 0.2%, CaCO_3_ 0.2%, pH 7.4	1000-L fermentor	52 h	65.34 U/mL	Site-directed mutagenesis (amino acid point mutations)		Yuan et al. (2024b)
*Streptomyces mobaraensis* smY2019-3C Plasmids pET-22b (+) and *Escherichia coli* BL21 (DE3)	Glycerol, peptone, yeast extract, K_2_HPO_4_, MgSO_4_, pH 7.2	250-mL flasks	96 h	40.00 U/mL	Regulation of TG-activating protease (TAP) activity in *S. mobaraensis*		Yin et al. (2021b)
*S. hygroscopicus* WSH03-13 *Streptomyces lividans* TK24 pTGM	Glycerol 2%, peptone 2%, yeast extract 0.5%, MgSO_4_ 0.2%, K_2_HPO_4_ 0.2%, KH_2_PO_4_ 0.2%, CaCl_2_ 0.1%	30-mL of medium	42 h	5.73 U/mL	Promoter engineering and codon optimization		Liu et al. (2016)
*Pichia pastoris* + pro-mTG pGAP9-pro and pPICZα-rDNA-mtg expression vectors	BMMY medium containing 1% methanol	1-L flasks	72 h	1.41 U/mL	Gene integration into the *P. pastoris* genome (methanol vector, AOX1)		Song et al. (2019)
*Pichia pastoris* GS115 Plasmid pPIC9K-TG	BMMY medium containing 5% methanol	Flasks	96 h	0.70 U/mL	Heterologous expression, integration (pPIC9K)		Yang and Zhang (2019)
*Escherichia coli* Chimera TEV–pro-MTG BL21(DE3)	Tryptone, yeast extract, glycerol, K_2_HPO_4,_ KH_2_PO_4_	250-mL flasks	48 h	22.7 U/mL	Recombinant cloning, chimeric gene, expression		Sato et al. (2020)
*Escherichia coli* BL21(DE3) Plasmid pET28a-X9tgl	Pepton 1%, yeast extract 0.5%, NaCl 1%	1-L flasks	4 h	5.73 U/mg	Construction of the pET28a- X9tgl plasmid, cloning	Zou et al. (2024)	
*Corynebacterium glutamicum* YDK010 Plasmid pPSPTG51	Glucose 6%, MgSO_4_ 1%, (NH_4_)SO_4_ 3%, KH_2_PO_4_ 0.15%, FeSO_4_·7H_2_O 0.001%, MnSO_4_·4H_2_O 0.001%, thiamine hydrochloride 450 µg/L, biotin 450 µg/L, dl-methionine 0.015%, CaCO_3_ 0.5%, pH 7.5	-	72 h	26 U/mg	Plasmid transformation; stable recombinant expression	Date et al. (2004)	
*Corynebacterium glutamicum* ATTC13869 T7C/E58C mutant	Glucose 6%, MgSO_4_ 0.1%, (NH_4_)_2_SO_4_ 3%, KH_2_PO_4_ 0.15%, FeSO_4_·7H_2_O 0.001%, MnSO_4_·4H_2_O 0.001%, 450 µg of thiamine hydrochloride, 450 µg of biotin, dl-methionine 0.015%, CaCO_3_ 5%, pH 7.5	50-mL flasks	48 h	27 U/mg	Rational and random mutagenesis	Yokoyama et al. (2021)	
*Streptomyces mobaraensis* ARTP mutation Sm5-V1	Glicerol 2%, tryptone 2%, corn steep powder 0.55%, yeast extract 0.5%, MgSO_4_ 0.2%, K_2_HPO_4_·3H_2_O 0.262%, CaCO_3_ 1%, pH 7.2	250-mL flasks	24 h	5.85 U/mL	Atmospheric and room temperature plasma mutagenesis (ARTP)	Jiang et al. (2017)	
*Yarrowia lipolytica* Po1h Vectors pINA1296/pro-TG, pINA1297/pro-TG	Glucose 2%, yeast extract 0,132%, NH_4_Cl 0.132%, KH_2_PO_4_ 0.032%, MgSO_4_·7H_2_O 0.024%, thiamine 0.0033%, pH 6.0	3-L fermenter	140 h	5.3 U/mL	Genomic integration, homologous recombination	Liu et al. (2015)	
*Streptomyces mobaraensis* TX1 Sm2−1 mutant	Glycerol, yeast extract, peptone, MgSO_4_·7H_2_O, K_2_HPO_4_·3H_2_O, (NH_4_)_2_SO_4_, pH 7.4	Flasks	32 h	37.51 U/mL	Ultraviolet (UV) light mutagenesis	Huang et al. (2022b)	
*Bacillus subtilis* 168 (ATCC 33712)	Corn starch 0.25%, peptone 2%, urea 0.08%, K_2_HPO_4_·3H_2_O 0.326%, KH_2_PO_4_ 0.254%, MgSO_4_ 0.092%, NaCl 0.3%, sucrose 3.5%, pH	Flasks	48 h	29.6 U/mg	Natural transformation of *B. subtilis*, homologous recombination	Mu et al. (2018)	
Gen TG from *Zea mays* (tgz) *Pichia pastoris* GS115 Vectors pPIC9K	Glycerol 1%, yeast extract 1%, peptone 2%, potassium phosphate 100 mM, yeast nitrogen base without amino acids 1.34%, biotin 4·10^–5^%, pH 6.0 Glycerol	Flasks	96 h	0.889 U/mL	Recombinant expression	Li et al. (2013)	
*Escherichia coli* BL21Gold(DE3) Plasmid rMTG(S2P)	Tryptone/peptone 1%, NaCl 1%, yeast extract 0.5%, ampicillin 0.01%, glucose based minimal medium containing 0.2%, Na_2_SO_4_, (NH_4_)_2_SO_4_ 0.268%; NH_4_Cl 0.05%; K_2_HPO_4_ 1.46%; Na_2_HPO_4_·2H_2_0 0.4%; (NH_4_)_2_-citrate 0.1%; MgSO_4_·7H_2_O 0.05%; thiamine 0.001%, 3 mL/L trace element solution were used for cultivation: CaCl_2_ 0.05%; ZnSO_4_·7H_2_O 0.018%; MnSO_4_·H_2_O 0.01%; Na_2_–EDTA 1%, FeCl 0.835%, CuSO_4_·5H_2_O 0.016%, CoCl_2_·6H_2_O 0.018, pH 7.5	20-L bioreacor	16 h	46.1 U/mg	Protein engineering (S2P mutation), recombinant expression	Sommer et al. (2011)	
*Corynebacterium ammoniagenes* ATCC6872 Plasmid pPKPTG1	Glucose 12%, MgSO_4_·7H_2_O 0.1%, (NH_4_)_2_SO_4_ 3%, KH_2_PO_4_ 0.15%, FeSO_4_·7H_2_O 0.001%, 0.001 g MnSO_4_·4H_2_O 0.001%, 450 µg thiamine hydrochloride, 450 µg biotin, g dl-methionine 0.015, 0.02% total nitrogen of soybean, pH 7.2	1-L fermentor	71 h	2.5 mg/L (protransglutaminase)	Recombinant expression, pro-TG secretion	Itaya and Kikuchi (2008b)	
*Escherichia coli* BL21Gold (DE3) Plasmid pET20b	Glucose 0.045%, lactose 0.12%, glycerol 0.45%, KH_2_PO_4_ 22.5 mM, Na_2_HPO_4_ 17.3 mM, MgSO_4_ 1.8 mM, pH	100-mL flasks	24 h	1.46 U/mL	Soluble expression of a pro-TG	Marx et al. (2007)	

In the study by Date et al. (2004), transglutaminase was produced by the *Corynebacterium glutamicum* YDK010 strain using the pro-region derived from the *S. mobaraensis* IFO13819 and *S. cinnamoneus* IFO12852 strains. It is worth emphasizing that the activity of purified transglutaminase using the pPSPTG51 plasmid was similar to that of native MTG and was 26 U/mg. The study conducted by Kikuchi et al. (2003) showed that *Corynebacterium glutamicum* ATCC 13032 containing the pVKTG3 plasmid was capable of efficiently producing transglutaminase derived from *Streptomyces mobaraensis* CICC 11018. Furthermore, this plasmid enabled the expression of sequences encoding the transglutaminase precursor protein (SAM-P45) and a serine protease with a structure similar to subtilisin, which was responsible for converting the proenzyme to its active form. It is worth noting that after 140 h of cultivation, the authors obtained an enzyme concentration of 142 mg/L. Similar studies were conducted using *Corynebacterium ammoniagenes* ATCC 6872. This strain contained the recombinant pPKPTG1 plasmid, which included the gene encoding protransglutaminase (pro-MTG). During 71 h of cultivation in a jar-type bioreactor, a protransglutaminase concentration of approximately 2.5 g/L was achieved (Itaya and Kikuchi 2008).

Alvarez et al. (2020) conducted studies to clone the gene encoding the Atlantic cod transglutaminase (AcTG-1) into a bacterial expression vector. They used it to transform protein expression in the *Escherichia coli* BL21 (DE3) strain. Biochemical characterization of the resulting transglutaminase showed that the enzyme retained activity in a temperature range from 0 to 65 °C, but it was inactivated entirely after 20 min of incubation at 70 °C. Furthermore, recombinant engineering yielded an enzyme with high catalytic activity at low temperatures. Liu et al. (2011) reported transglutaminase production using the same *E. coli* BL21 (DE3) strain after cultivation in Terrific Broth medium containing ampicillin. Transglutaminase derived from *Streptomyces hygroscopicus* strain WSH03-13 was secreted by an *E. coli* strain using a signal peptide specific to this enzyme. Transglutaminase activity was approximately 4.5 U/mL. In the study presented by Wang et al. (2025), the possibility of obtaining a genetic fusion construct was investigated by combining the pro-region of transglutaminase derived from *Streptomyces hygroscopicus* (proH) with the gene encoding TrxA. This approach aimed to improve the solubility and folding efficiency of the enzyme when expressed in *Escherichia coli* BL21 (DE3) cells. The resulting recombinant strain showed intracellular transglutaminase activity of 9.78 U/mL.

In subsequent studies using an expression system based on *Escherichia coli* BL21 (DE3), a strategy for cloning and expressing microbial transglutaminase derived from *Streptomyces mobaraensis* was presented (Javitt et al. 2017). The bacteria were cultured for 60 h at 25 °C in Terrific Broth medium with kanamycin. The concentration and activity of the obtained mTG were 120 mg/L and 1 U/mL, respectively. In the study conducted by Wan et al. (2017), it was found that expression of the transglutaminase gene derived from *S. hygroscopicus* H197 in a recombinant *E. coli* strain resulted in a transglutaminase activity of 0.69 U/mg. The obtained result improved compared to the transglutaminase activity produced by the native microorganism strain (*S. hygroscopicus* H197), which was only 0.54 U/mg.

Furthermore, the presented results indicate that using an expression system in *E. coli* can increase the enzyme’s production efficiency and catalytic activity. The use of recombinant bacterial strains offers the possibility of further optimization of culture conditions and genetic modifications. Such actions may lead to a transglutaminase with even greater activity and improved enzymatic properties.

Lin et al. (2008) expressed transglutaminase derived from *Streptomyces platensis* strain M5218 using a recombinant *Streptomyces lividans* strain JT46. Enzyme activity was 5.78 U/mL at an optimal temperature of 55 °C and pH 6 (grown in flasks). However, when cultivating in 5- and 250-L bioreactors, enzyme activities were 5.39 and 2.54 U/mL, respectively. In subsequent work, Liu et al. (2015) obtained protransglutaminase using a recombinant yeast strain, *Yarrowia lipolytica* Po1h. The authors used the pINA1297 (multicopy) vector, and before transformation, the gene responsible for kanamycin resistance was deleted from *Y. lipolytica*. The transglutaminase activity obtained in the deglycosylated N355Q variant was 35.0 U/mL. Interestingly, Li et al. (2013) transformed a plant transglutaminase fragment derived from common maize (*Zea mays*) into the *Pichia pastoris* GS11 strain. The recombinant strain was then cultured for 96 h at 28 °C in Buffered Methanol-Complex Medium (BMMY) with glycerol replaced by methanol. The resulting transglutaminase production was 4.4 mg/L, and the specific activity was 0.889 U/mg. In other studies, *Pichia pastoris* X33 (*Komagataella phaffii*), as an expression organism of pro-mTGase derived from *S. mobaraensis*, was able to produce transglutaminase at a level of approximately 37.640 U/L after 79 h of fermentation (Türkanoğlu Özçelik et al. 2019). Aqeel et al. (2024) used a single expression cassette containing two different promoters for the propeptide region derived from *S. mobaraensis* strain DSM 40847 and mature mTG, which led to the secretion of fully functional mTG in *Komagataella phaffii* strain GS115 (*Pichia pastoris*). The secretion efficiency of mTGase after yeast cultivation in a 5-L fermenter reached 47.96 U/mL.

The study by Yuan et al. (2024a, b) demonstrated the successful construction of the recombinant *S. mobaraensis* smL2020ΔTG strain. The newly constructed strain demonstrated high transglutaminase production efficiency at laboratory and pilot scales. During culture in flasks and in a 1000-L bioreactor, enzymatic activities were 56.43 and 63.18 U/mL, respectively. It is worth noting that the obtained results indicate the high scalability of the production system and the high potential for industrial use of the modified strain.

Another example of utilizing the biotechnological potential of microorganisms was the use of *Bacillus amyloliquefaciens* XCT-09. Subsequently, the gene responsible for transglutaminase production (X9Tgl) from the XCT-09 strain was cloned and extracted from *Escherichia coli* BL21 (DE3). The specific activity of the purified X9Tgl enzyme reached 5.73 U/mg (Zou et al. 2024).

Sommer et al. (2011) described the expression of a thermostable transglutaminase variant by the recombinant *E. coli* BL21 Gold (DE3) strain with the expression plasmid pCM203. In the produced transglutaminase variant, serine was replaced with proline at position 2, increasing the enzyme activity to 46.1 U/mg. Furthermore, the obtained and purified enzyme preparation was free of proteases that can cause protein degradation. Studies by Hirono-Hara et al. (2021) showed that the use of a gene derived from *Streptoverticillium mobaraense*, which encodes mTG, can be optimized and transformed into the *S. cerevisiae* BY4741 strain. Based on the results, the authors found that the transglutaminase activity of strain BY4741 with the TG gene was approximately 45 U/mL. In another study (Ye et al. 2024), plasma-mediated mutagenesis was used on the *Streptomyces mobaraensis* strain DSM40587. This method resulted in the smY2022 mutant, capable of producing mTG activity of approximately 41 U/mL. In a further step, the transglutaminase coding sequence in smY2022 was genetically modified. It was replaced with the optimized TGm2 sequence, which allowed the construction of a new strain: smY2022-TGm2. This modification resulted in a transglutaminase activity of 61.7 U/mL.

It is worth emphasizing that many intensive efforts are underway to obtain microbial transglutaminase with high enzymatic activity and increased biosynthetic efficiency. In this context, various strategies for optimizing the expression of the gene encoding transglutaminase play a particularly important role, encompassing genetic modification of producer strains and metabolic engineering, optimization of fermentation conditions, and the use of high-yield expression vectors. Due to the growing demand for biocatalysts with optimized properties, innovative biotechnological solutions are constantly sought. Therefore, culturing genetically improved microbial strains may be an important research direction for the industrial production of mTG.

## Potential health risks associated with using TG

Due to its potential properties, transglutaminase is widely used in the food industry (Vasić et al. 2023). As enzymology advances and this enzyme is increasingly used in numerous technological endeavors, concerns arise regarding the potential health risks associated with its use. This is related to the possibility of various immune reactions.

The influence of mTG on the development of autoimmune diseases (e.g., celiac disease) is one of the main research topics conducted by various research centers worldwide (Lerner and Benzvi 2021; Besser and Khosla 2023). Tissue transglutaminase (tTG) plays a key role in the course of this disease (Di Sabatino et al. 2012). It modifies gliadin peptides derived from gluten (Rauhavirta et al. 2016), forming structures recognized by the immune system as foreign. Furthermore, ingestion of gluten-related proteins, such as hordein from barley, secalin from rye, or avenin from oats, can trigger inflammatory reactions in the human body (Sharma et al. 2024). Gliadin is a potent immunotoxic agent that can disrupt the assembly of junctions in epithelial cells (Chander et al. 2018). It is worth noting that microbial TG exhibits functional similarity to tTG (Matthias et al. 2016). This may raise concerns regarding its possible influence on the development or intensification of autoimmune reactions. Some studies suggest that complexes of mTG with gluten proteins may form immunogenic structures (Dekking et al. 2008; Torsten and Aaron 2018), potentially recognized by antibodies against tTG (Matthias et al. 2016). Scientific reports presented by Lerner and Benzvi (2021) suggest that mTG, through its enzymatic properties, can catalyze the cross-linking reaction of gluten molecules, leading to the formation of protein complexes as new autoantigens. TG can deamidate some gluten peptides (Lorand and Iismaa 2019; Lerner et al. 2025), increasing their affinity for the Human Leukocyte Antigen class II (HLA-DQ2/DQ8) molecules (Heil et al. 2016). The consequence of these processes is the activation of T lymphocytes (Dekking et al. 2008) and the induction of an immune response in genetically susceptible individuals. mTG stimulates the human immune system to produce specific antibodies. The immunogenicity of these antibodies has been reported only in patients with celiac disease (Chander et al. 2018). Furthermore, mTG may increase intestinal permeability (Lerner et al. 2025; Camilleri 2025), which further facilitates the penetration of antigens and may increase the risk of food hypersensitivity (Bauer et al. 2025). Thus, TG may support the immune response, contributing to the development of celiac disease (Lerner and Matthias 2019) and other autoimmune diseases.

It is worth emphasizing that transglutaminase can form new covalent isopeptide bonds between proteins (Kieliszek and Misiewicz 2014). Some of the resulting protein complexes are resistant to proteases, bile acids, and reducing agents, which leads to a prolonged half-life in the intestinal environment (Lerner and Matthias 2019). Consequently, they may have a longer effect on the immune system and perform harmful biological functions. Furthermore, these processes may lead to new epitopes, i.e., protein fragments recognized by the immune system (Matthias et al. 2016). Such epitopes can trigger allergic reactions (Lerner et al. 2023). Furthermore, mTG may influence the bioactivity of peptides formed during digestion. Some proteins may be degraded into peptides that exhibit proinflammatory effects (Lerner et al. 2017). Furthermore, some authors report a possible link between the activity of various transglutaminase isoforms and the development of certain neurodegenerative disorders (Lesort et al. 2000). In the context of Alzheimer’s disease and Huntington’s disease (HD), it has been observed that transglutaminase may participate in pathological protein aggregation (Lerner et al. 2023; Beninati et al. 2024), leading to the formation of insoluble protein complexes in neural tissue. Such complexes can disrupt cellular homeostasis (Lerner and Benzvi 2021) and contribute to neurotoxicity.

What’s also important is that, legally, transglutaminase is not classified as an allergen. However, the Food Allergen Labeling and Consumer Protection Act (FALCPA), which regulates the labeling of allergens in food, also does not classify this enzyme as an allergen. According to information provided by Lambré et al. (2024), food products to which transglutaminase is added are typically heat-treated, which inactivates the enzyme and prevents it from forming cross-linked products that can cause allergies. It is worth noting that some people may be allergic to certain proteins (e.g., milk or eggs). Such consumers may exhibit increased reactivity to food products in which mTG has been used to modify the protein structure.

Another important aspect related to the safety assessment of mTG use is the currently available scientific data, which indicate that this enzyme does not exhibit mutagenic effects (Bernard et al. 1998). However, Lerner and Benzvi (2021) report that individual declarations that transglutaminase is non-toxic, safe, non-allergic, and non-immunogenic to human health are contradictory to various scientific publications. The authors believe that TG or its cross-linked complexes may be toxic. Long-term toxicological studies and clinical trials in humans should confirm an appropriate assessment of the safety of transglutaminase. Unfortunately, the number of epidemiological studies conducted in humans involving chronic dietary mTG consumption is limited. As Amirdivani et al. (2018), the effect of microbial transglutaminase in food products is controversial regarding its safety in individuals with autoimmune diseases such as celiac disease (CD). In publications presented by Gerrard and Sutton (2005) and Lerner and Matthias (2015), the authors demonstrated that wheat or gluten treated with mTG has immunogenic properties, induces antibodies, and generates stimulating T cell epitopes involved in the development of celiac disease. Similar results were obtained by Zhou et al. (2017). In their study, the authors found that using mTG can increase the immunogenicity of gliadin peptides, leading to their recognition by T lymphocytes in individuals with celiac disease. It is also imperative that the potential immunogenic effect of transglutaminase complexes may be induced by cross-linking this enzyme with proteins structurally similar to gluten (Lambré et al. 2024). These processes may result in immune reactions in people with celiac disease. Treppiccione et al. (2022) presented completely different data on transglutaminase. The authors stated that mTG has not yet been shown to have pathogenic activity. Furthermore, they report that using mTG effectively reduced the inflammatory immune response to gluten in Crohn’s disease. Moreover, the transamidation process catalyzed by mTG is characterized by selectivity for glutamine residues present in immunogenic gliadin peptides. These actions limit deamidation by tissue transglutaminase (tTG) and prevent the formation of tTG–gliadin complexes (Treppiccione et al. 2022). Studies presented by Zhu et al. (2019) showed that using TG as a new tofu coagulant reduces its potential allergenic properties. Furthermore, Zhang et al. (2025) found that transamidated gliadin (GM) formed with TG is characterized by reduced toxicity in the context of celiac disease. These results, which reflected the intestinal environment typical of this disease, were confirmed in bone marrow–derived dendritic cell models and in the BALB/c mouse cell model. These results suggest that the transamidation process may be a promising and safer therapeutic strategy for the treatment or prevention of celiac disease symptoms. According to information provided by Bradauskiene et al. (2021), lactic acid bacteria can help degrade immunotoxic gluten peptides produced by transglutaminase. Another aspect is that short-chain fatty acids produced by lactic acid bacteria have anti-inflammatory potential (Gill et al. 2018), the potential to restore intestinal barrier function, and the potential to modulate the function of regulatory T lymphocytes in the intestinal mucosa (Kim 2021).

In summary, there is an urgent need to clarify the full impact of transglutaminase on the human body. Despite numerous declarations regarding the safety of transglutaminase in industrial applications, there is currently a lack of clear and direct evidence confirming or refuting its potential immunogenicity. The wide variety of opinions regarding the functioning of this enzyme can cause misunderstanding and confusion among individual consumers and food technology professionals.

## Legal regulations regarding the use of transglutaminase

Transglutaminase is an enzyme primarily used as an adhesive for cross-linking proteins and other molecules during food processing (Lerner and Benzvi 2021). Examples of this process include the joining of meat fragments (Kaić et al. 2021) and the cross-linking of food (Wang et al. 2025). Transglutaminase is considered an auxiliary substance in technological processes that does not fulfill the function of a final ingredient. It is worth emphasizing that the information provided by the Codex Alimentarius document Guidelines on Substances Used as Processing Aids (CAC/GL 75–2010) states that this enzyme, as an auxiliary substance used in industrial processing, does not perform an independent technological role in the final product. Consequently, such activities exempt food producers from the obligation to list this enzyme on the product label.

In the European Union, transglutaminase is subject to formal regulations set out in Regulation (EC) 1331/2008 (European Commission 2008a), which outlines procedures for the safety assessment and authorization of food additives, enzymes, and flavorings, and in Regulation (EC) 1332/2008 (European Commission 2008b), which concerns enzymes in food. It is worth noting that the European Food Safety Authority (EFSA) has recently issued detailed opinions on transglutaminase obtained from the non-genetically modified *Streptomyces mobaraensis* strain M2020197 (Lambré et al. 2024). The presented document contains detailed opinions on, among other things, the production, potential risk, toxicological data, and allergenicity of the presented transglutaminase. In another document, the EU stated that the food enzyme γ-glutamate α-glutamyltransferase produced by the non-genetically modified *S. mobaraensis* AE-BTG strain does not raise safety concerns (Zorn et al. 2024). EU food labeling regulations state that transglutaminase used as a “*processing aid*” generally does not have to be listed in the ingredients list. The only exception may be a justified risk of allergen transmission. Therefore, when using an enzyme preparation containing any allergenic ingredients, food manufacturers are asked to clearly label these ingredients on the finished products (Giosafatto et al. 2018). According to the documents presented in Regulation (EU) No 1169/2011 (European Commission 2011), food products in which slices of meat or fish have been formed from smaller parts joined together, regardless of the use of transglutaminase, should be marked on the label as “*formed meat*” or “*formed fish*.”

In the USA, transglutaminase has been recognized as a GRAS (Generally Recognized As Safe) substance since 1998 (Romeih and Walker 2017), meaning that independent expert opinions and documents confirm its safety. It is worth emphasizing, however, that producers of this enzyme declare its non-toxicity, safety, and lack of allergenic properties; this information is not always consistent with data available in the scientific literature (Lerner and Benzvi 2021). At the same time, the Food Safety and Inspection Service (FSIS) has issued an opinion on the use of this enzyme as a “*binder*” in some standardized meat and poultry products. Furthermore, transglutaminase is also called “*meat glue*” (Bub et al. 2013). It is worth noting that the labeling requirement for all products manufactured using transglutaminase is still in effect. The products presented must be labeled as “*formed*” or “*restructured*” meat (Lerner et al. 2025), and the ingredient must be listed on the label (Bub et al. 2013). In Canada, enzyme regulations are specified in the *List of Permitted Food Enzymes*, maintained by Health Canada and the Canadian Food Inspection Agency (CFIA) (Nunes et al. 2018; Sahu et al. 2023). In 2025, Health Canada updated its list of food additives to include transglutaminase produced by *Streptoverticillium mobaraense* strains S-8112 and M2020197. This enzyme is included on this list with detailed information on its source and permissible technological uses, which means it is officially approved for use in the food industry.

In China, transglutaminase is approved for use in the food industry (Giosafatto et al. 2018). At the same time, according to information provided by the China National Center for Food Safety Risk Assessment, consultations on several food additives have been underway since September 26, 2024, including transglutaminase produced microbiologically by *Bacillus licheniformis* and *Streptomyces mobaraensis* (China National Center for Food Safety Risk Assessment 2024).

In Australia and New Zealand, transglutaminase is considered a technological enzyme by Food Standards Australia New Zealand (FSANZ). In 2024, the use of transglutaminase from the genetically modified bacterium *Bacillus licheniformis* as a processing aid in food production was permitted (Food Standards Australia New Zealand 2024). The use of enzymes derived from natural strains of *Streptomyces mobaraensis* is also permitted (Food Standards Australia New Zealand 2021). Current regulations do not require producers to declare this enzyme on the product label. However, this does not exclude the need to label a product as “*formed meat*” when the meat was formed from fragments linked enzymatically. In Japan, the food additive transglutaminase is regulated by the Food Safety Commission of Japan (FSCJ). In 2024, an enzymatic preparation produced by the strains *Bacillus licheniformis* JPBL015 (Food Safety Commission of Japan 2024a) and *Streptomyces mobaraensis* TGG-1 (Food Safety Commission of Japan 2024b) was approved for use. Moreover, a similar situation is observed as in the EU, where transglutaminase is also classified as a “*processing aid*” and is usually not included in the list of ingredients. In Japan, it is recommended that manufacturers analyze the use of this enzyme in accordance with national guidelines and declare its presence in cases of technological justification. In Brazil, despite less unified regulatory systems, transglutaminase is also allowed as a “*processing aid*” (Rosseto et al. 2024). Moreover, in some cases there is an obligation to inform consumers that the food product was formed from combined pieces of raw material. In Brazil, the commercial use of transglutaminase is concentrated in meat restructuring processes (Beisembayeva et al. 2025), in the dairy beverage sector (De Abreu Costa et al. 2022), and in applications involving plant-based raw materials (e.g., soy-based) (de Góes-Favoni and Bueno 2014; dos Santos et al. 2023).

In summary, food production products containing TG as an excipient may be labeled. The scope and form of this obligation depend on the legal regulations in force in a given country or geographic region. Furthermore, such information about the composition of food products is crucial for consumers, as it allows individuals struggling with food allergies to make informed choices about specific foods.

## Potential applications of transglutaminase in the food industry

Microbial transglutaminase is widely used in the food industry. This enzyme can catalyze protein cross-linking reactions (Wang et al. 2025), leading to stable cross-links between molecules (Xu et al. 2025). Proteins constitute a key structural component of food products (Tolstoguzov 1991), determining their viscosity and water retention capacity (Gaspar and De Góes-Favoni 2015). Consequently, there is growing interest in food technology for methods that modify and improve protein functional properties. These processes result in products characterized by optimal quality parameters, which play a key role in consumer choice.

Transglutaminase has a variety of applications in food (Table 4), including improving texture (Iličić et al. 2013) and extending the shelf life of products. Furthermore, this enzyme enhances the consistency of meat products and increases food elasticity (Kazemi Kheirabadi et al. 2025). According to Turan et al. (2025), microbial transglutaminase was first used in the food industry in Japan. The enzyme produced reconstituted products, exemplified by kamaboko and fish paste. Noting the extensive potential for utilizing transglutaminase’s functional properties, the remainder of this article is devoted to analyzing examples of its use in various food industry sectors.

**Table 4:** Examples of the use of transglutaminase in various industrial sectors

Industry	Protein substrates	Technological effects	Enzyme dose	References
Meat processing	Meat	Fragment bonding, texture, hardness	< 15 U/g	Sun and Arntfield (2011); Santhi et al. (2017); Yang and Zhang (2019)
Fish	Fish proteins (myofibrillar)	Fragment bonding, improved gelation and cohesion	0.1–9.0 U/g	Moreno et al. (2010); Chanarat et al. (2012); Taghi Gharibzahedi et al. (2018)
Dairy	Casein, whey	Stabilization, reduced syneresis, elasticity, stability	0.5–50.0 U/g	Farnsworth et al. (2006); Gauche et al. (2010); De sá and Bordignon-Luiz (2010); Tsevdou et al. (2013); Hovjecki et al. (2021); Velazquez-Dominguez et al. (2023)
Bakery	Gluten, plant proteins	Influences dough volume, structure, and texture	1.0–120 U/g	Rossi Marquez et al. (2014); Dłuzewska et al. (2015); Ouyang et al. (2021)
Plant products	Soy, peas, cereals	Improvement of texture and cohesion of analogs	0.2–8.0 U/g	Wen et al. (2022); Zimoch-Korzycka et al. (2024)

### Meat industry

Transglutaminase plays a fundamental technological role in the meat industry. It is the most commonly used enzyme in restructured meat products and analogues (Cheng et al. 2024). Furthermore, transglutaminase contributes to increasing the commercial value of meat products (Santhi et al. 2017) made from smaller raw material components, thanks to its ability to cross-link proteins (Wang et al. 2025). Such processes lead to their permanent connection and a several-fold increase in the hardness of the final product (Kieliszek and Błażejak 2017; Fatima and Khare 2021). Interestingly, TG is also involved in the production of certain types of reduced-fat meat (Kieliszek and Błażejak 2017; Yong et al. 2020), and plays a role in improving the structural properties of sausages by giving them a uniform and stable shape (Giosafatto et al. 2018).

Current research using mTG in the meat industry focuses on adapting production technology to current nutritional trends and growing consumer expectations. These aspects are fundamental to obtaining foods with increased nutritional value and health benefits. When using mTG for meat reconstruction, special attention must be paid to the fat content, which can negatively impact meat binding (Sorapukdee and Tangwatcharin 2018). Sorapukdee and Tangwatcharin (2018) demonstrated that combining raw beef with up to 17% fat content with 1% MTG (Activa TG-B preparation) increased meat tenderness and improved the sensory quality of the reconstructed steak (Duarte et al. 2019). Zhu et al. (1995) found that using transglutaminase to produce hamburger meat or dumpling fillings increased elasticity and improved consistency, flavor, and aroma. Adding this enzyme to products such as hams and cold cuts improved texture and increased tear strength (Alves et al. 2021; Lee and Chin 2022). This effect is due to forming bonds between glutamine and lysine in meat proteins (Ahhmed et al. 2009). Lee and Chin et al. (2011) observed that the presence of TG in pork ham provides higher tensile strength. The authors concluded that this enzyme provides adequate binding capacity for restructured meat products. Recent studies (Li et al. 2018; Erdem et al. 2020) quantified these effects: adding 1% mTG to beef increased gel strength by 35%, while hardness increased by 20%, and cohesiveness by 15% compared to untreated controls. It is worth noting that microbial transglutaminase allows for producing stable gels (Chang et al. 2023). According to Li et al. (2018), the efficiency of TG can stabilize a gel with higher porosity at appropriate concentrations, which can immobilize water more effectively. Introducing transglutaminase to meat products can promote the aggregation of myofibrillar proteins, thus improving protein gelation while reducing salt consumption (Niu et al. 2025). Furthermore, through covalent cross-linking of proteins, transglutaminase can effectively influence the formation of a gel matrix from meat proteins, even under low salt conditions (Feng et al. 2018). TG can improve the gelling properties of meat products induced by adding antioxidant substances. This enzyme increases the hydrophobicity of the protein structure by limiting the formation of disulfide bridges between myofibrillar proteins (Ren et al. 2024). Furthermore, TG can enhance meat products’ gelling and water-fat binding capacity (Seighalani et al. 2016). Protein gelation is an important functional property that modifies food texture (Moreno et al. 2020). Gaspar and Góes-Favoni (2015) described the differential gelling properties of proteins in different types of meat (e.g., beef, poultry). The authors found that differences in the number and spatial accessibility of lysine and glutamine residues influence the effectiveness of this enzyme. In beef, gel breaking force reached 0.067 × 10 N/m^2^ at 1% TG, while chicken required 0.047 × 10 N/m^2^ to achieve similar structural integrity, highlighting species-specific responses (Ahhmed et al. 2009). Studies presented by Feng et al. (2024a, b) demonstrated that the simultaneous use of transglutaminase and κ-carrageenan can significantly increase the viscosity and improve the viscoelastic properties of raw meat batter, leading to the formation of a stiffer network structure. In another study, Feng et al. (2024a, b) found that the degree of cross-linking of myofibrillar protein gels gradually increased with increasing transglutaminase concentration. Similar results were obtained by Ahhmed et al. (2009). The authors demonstrated that the breaking strength of raw meat increased in direct proportion to the amount of added transglutaminase. However, the elasticity of chicken meat was significantly lower than that of beef samples. These differences are due to the chemical and physiological properties of these two types of meat. Jira et al. (2017) demonstrated that increasing TG concentration improved the sensory evaluation of dry-cured, formed ham samples. The use of transglutaminase in the production of beef, pork, and poultry meatballs demonstrated a significant effect on the textural properties of these meat products. Samples containing TG were characterized by a harder and more uniform gel structure. Furthermore, improved parameters such as elasticity and cohesiveness were observed compared to control samples. Importantly, meat products with added transglutaminase scored higher in sensory analysis than their counterparts without the enzyme. For example, adding 1% TG to turkey meatballs, relative to the no-enzyme control, increased hardness from 89.12 to 100.84 N and cohesiveness from 0.36 to 0.39, as measured by texture profile analysis (Erdem et al. 2020). A study conducted by Canto et al. (2014) showed that the addition of TG (1%) and salt substitutes (0.375% KCl and MgCl_2_) increased juiciness and achieved the lowest hardness of restructured caiman steaks. Another study (Li et al. 2018) found that adding TG significantly increased gel strength, especially when using pork and silver carp in a 7:3 ratio.

Transglutaminase also enables the production of restructured meat by bonding small pieces together. A system has been developed that allows for the bonding of meat using both transglutaminase and caseinate (Carballo et al. 2006). Transglutaminase combined with caseinate becomes viscous, creating a glue that binds various food products. This method allows for creating large portions of meat using smaller pieces of these raw materials. Quantitative evaluation showed that sausages produced with the addition of 0.1% TG and 0.5% caseinate were characterized by 18% higher chewiness and 12% greater elasticity than sausages with TG alone (Colmenero et al. 2005). Therefore, the authors believe that caseinate is a suitable substrate for TG, facilitating cross-linking between meat protein and caseinate molecules, which favors the formation of a much more stable gel matrix. The resulting product retains conventional meat’s flavor, texture, and appearance (Kieliszek and Błażejak 2017). This method reduces losses in meat production by using transglutaminase to combine fragments that would otherwise be unsuitable for sale. Kuraishi et al. (1997) suggest that the addition of transglutaminase (0.05–0.1%) in combination with sodium caseinate (0.5–1.0%) allows for the production of restructured products. These conclusions were confirmed by studies conducted by Colmenero et al. (2005). The authors demonstrated that combining TG with caseinate resulted in harder, more elastic, and chewier sausages with low sodium content and walnuts. These products demonstrated better water and fat binding properties than those with TG alone. Research by Yang and Zhang (2019) showed that transglutaminase improved the chewiness, hardness, and quality of restructured pork. Research by Kilic (2003) showed that adding mTG, both with and without sodium caseinate, induced protein cross-linking in chicken döner kebab meat. However, enzyme activity was significantly higher when it was used in combination with sodium caseinate.

In the production of sausages and cold cuts, it is possible to use raw materials of lower technological value, such as mechanically deboned meat (Cavenaghi-Altemio et al. 2018), and additives such as skimmed milk powder, soybean meal, or wheat flour (Kieliszek and Błażejak 2017; Al-Temimi et al. 2024). These raw materials can then be subjected to enzymatic modifications. The role of transglutaminase is to impart the appropriate texture characteristic of meat to the final products. Such processes allow for producing products with properties similar to those of high-quality meat products (Kieliszek and Błażejak 2017; Duarte et al. 2019).

The production of meat analogues is a response to the growing demand for alternative food products for vegetarians and consumers seeking substitutes for animal products (Miwa 2020). The production of meat analogues poses a significant technological challenge for the food industry, primarily due to difficulties in replicating animal products’ texture and sensory profile. Current research focuses on using plant proteins, such as pea protein, in combination with transglutaminase (Dong et al. 2023; Yu et al. 2025). Such efforts aim to improve structural properties and achieve a texture similar to meat products (Maningat et al. 2022; Leelapunnawut et al. 2022). For example, the study presented by Moreno et al. (2020) examined the effect of TG on the structural quality of pea protein isolate. The study demonstrated that the mTG enzyme significantly enhanced the structural quality of pea protein isolate (PPI)–aqueous dispersion gels (high Q-factor) at concentrations of 20% and 23% PPI. It is worth noting that these gels are more suitable for achieving consistent gels as bases for meat and seafood analogues. In summary, such gels may represent an interesting alternative for industry as a base for producing meat analogues. A modern approach to producing meat analogues is the use of 3D printers. The study by Wen et al. (2022) aimed to develop a beef analogue that mimics the textural properties of the raw material using 3D printing and transglutaminase. The use of transglutaminase (TG) at a level of 4 U/g protein resulted in printed meat analogues with a smooth surface and high hardness. In addition to developing an appropriate recipe, the study assessed various thermal processing methods for the resulting product. It was found that meat analogues baked at 170 °C had textural properties similar to beef. As a result, technological recommendations were developed for producing this type of meat analogues. As reported by Zimoch-Korzycka et al. (2024), the addition of a higher TG concentration and extended incubation time significantly improved the protein content and textural properties of poultry meat analogues obtained from wheat and pea fiber. dos Santos et al. (2023) reached slightly different conclusions. The authors demonstrated that adding TG reduced the thermal processing efficiency of hybrid sausages made from rice protein. Furthermore, textural parameters significantly increased in the presence of the enzyme. In the case of hybrid sausages based on soy protein, only cohesiveness and chewiness were improved. According to Wen et al. (2022), increased TG content improved the rheological properties of raw meat analogues based on bean protein. The authors found that using the enzyme at a dose of 4 U/g protein led to meat analogues characterized by a smooth surface and a significant increase in hardness. The study by Yu et al. (2025) showed that TG significantly affected the functional properties of meat analogues obtained from pea protein, corn flour, and wheat gluten. The use of the enzyme contributed to increased hardness of the extrudates and brightened their color.

It is worth emphasizing that there are many benefits to using microbial transglutaminase in meat processing. The most significant is the ability to rationally utilize waste materials by producing restructured products. These possibilities open up new perspectives for using transglutaminase in various aspects of the meat industry.

### Fish processing industry

Transglutaminase is widely used in fish processing due to its protein-binding properties (Huang et al. 2023). This enzyme enables the effective binding of various fish fragments (Kaufmann et al. 2012), which improves product structure and consistency. Transglutaminase benefits the physicochemical properties of fish products (Alves et al. 2021), which is important for their quality and stability. Transglutaminase can extend shelf life (Yerlikaya et al. 2017). Furthermore, this enzyme allows for producing a product with increased value (Tokay et al. 2021), which is highly functional and has water-retaining properties (Kaewprachu et al. 2017). Such enzymatic properties give processed seafood a firm and elastic texture (Yerlikaya and Gokoglu 2024). Examples of food products obtained using TG include fish balls and sticks, surimi, products imitating crab meat and shark fins, kamaboko (fish cake), and Japanese fish paste (Kieliszek and Błażejak 2017). Transglutaminase is also used in Japan to process, among others, salted pollock roe (Chen et al. 2019), cod (Kuraishi et al. 2001), and frozen seafood, e.g., shrimp (Tammatinna et al. 2007; Jiang et al. 2023). The use of transglutaminase in the fish industry began with the production of surimi (fish paste) in Japan (Kuraishi et al. 2001; Turan et al. 2025). Studies described by Vácha et al. (2006) showed that a small addition of TG (0.1%) to fish material increased gel strength from 45 to 54%. Similarly, using TG at 0.3–0.5% in combination with salt increased gel firmness by 60–75% and water retention by 10–12%, demonstrating practical improvements in textural properties (de Góes-Favoni and Bueno, 2014). Using the enzyme at a concentration of 0.5% in combination with NaCl (1.0%) improved the product firmness by 71% compared to the control sample. Furthermore, adding TG and NaCl increased the water content from 53 to 64.4% (de Góes-Favoni and Bueno 2014). The study by Pérez-Mateos et al. (2002) determined the effect of TG addition on the gelation properties of frozen squid. Using 0.02% transglutaminase with protease inhibitors increased the frequency of covalent bonds, significantly improving gel elasticity. According to Kim et al. (2024), adding 0.37% TG significantly increased the gel strength of fish patties. At the same time, using TG in the cross-linking process at 4 °C promoted the formation of a more uniform and stable fish gelatin emulsion (Qi et al. 2025). As reported by Fang et al. (2025), TG improves the gelling properties of fish gelatin by catalyzing the binding reaction between the ε-amino group on lysine residues and the γ-carboxamide group on glutamine residues in the gelatin molecule. For example, in subsequent studies (Alves et al. 2021), it was found that adding TG beneficially reduced losses during the cooking process of fish ham. Simultaneously, using this enzyme increased the hardness, cohesiveness, and chewiness. Importantly, TG improved the ham’s tensile strength. Similar results were obtained by Monteiro et al. (2015). The authors noted that using transglutaminase with tilapia pieces improved the sensory and textural properties of the resulting steak. Another study (Zhang et al. 2022) found that adding TG can improve the quality of frozen gel from ground southern cod (*Patagonotothen ramsayi*). It is worth noting that in another study (Shan et al. 2023), TG reduced bitterness and increased umami flavor content in the hydrolyzed skeleton of Alaska pollock. This was due to a reduction in the content of hydrophobic amino acids and an increase in their sweet counterparts. Furthermore, UV spectral analysis showed that transglutaminase cross-linking of hydrolysates induced changes in the protein structure, resulting in cross-linking of peptides and amino acids. In another study (Kunnath et al. 2021), using ground pink perch samples with TG revealed a significant effect of this enzyme on textural properties, especially in inducing gel strength. Huang et al. (2022a, b) noted another application of transglutaminase. Adding 1% TG to yellow perch resulted in an increase in hardness and elasticity by approximately 20% and an improvement in moisture content by approximately 10% compared to the control samples (Altan et al. 2024; Kunnath et al. 2021). The study found that adding 1.0% TG to the curing solution improved the texture of yellow perch (*Pseudosciaena crocea*), even after heat treatment. Furthermore, the authors observed increased hardness, chewiness, and moisture content in the cured perch. In a separate study (Altan et al. 2024), TG increased the elasticity, hardness, and shear strength parameters of rainbow trout (*Oncorhynchus mykiss*) burger patties compared to the control sample. These data are consistent with the results obtained by Tzikas et al. (2018), where TG had a positive effect on elasticity in restructured horse mackerel (*Trachurus mediterraneus*) products. In the study by Corapci et al. (2024), the addition of the enzyme influenced the color values. Also, it contributed to improving the sensory quality of reconstituted frozen rainbow trout patties.

### Baking industry

The most important benefits of adding transglutaminase include improving the rheological properties of the dough, i.e., its stability, elasticity, springiness, and water adsorption, as well as extending the shelf life of the products (Collar and Bollaín 2004; Scarnato et al. 2017; Fatima and Khare 2018; Šmídová and Rysová 2022). An example of using TG can be found in the article by Long et al. (2025). The use of this enzyme had a positive effect on the quality of high-fiber crackers enriched with soybean hulls. Furthermore, the resulting crackers met nutritional expectations and demonstrated sensory acceptability. Similar results were obtained by Nguyen et al. (2025). The authors demonstrated an improvement in the texture of high-fiber crackers after adding mTG, which translated into a higher level of consumer acceptance. Furthermore, adding transglutaminase to bakery products effectively improves the quality of fresh bread (Ogilvie et al. 2021) and biscuits (Rossi Marquez et al. 2014). Zhou et al. (2017) highlighted another potential application of transglutaminase (TG), pointing to its role in reducing the allergenicity of proteins present in wheat flour, which may be a promising option for supporting the treatment of conditions such as celiac disease. According to Kieliszek and Błażejak (2017) and Huang et al. (2008), the addition of transglutaminase strengthens the structure of the dough, which can be used in frozen products before baking. This results in products less susceptible to damage associated with freezing. Furthermore, according to Scarnato et al. (2017), TG could form isodipeptide bonds in the gluten fraction, leading to protein aggregates. These processes resulted in an improved structure and aroma of sourdough bread. Moreover, some peptides released from gluten by TG can modulate the microflora of bread, potentially extending its shelf life (Duarte et al. 2019; Gharibzahedi and Altintas 2024). Furthermore, using TG can increase bread volume and reduce its hardness and stringiness (Redd et al. 2024). The effectiveness of this process varies depending on the flour composition. However, protein cross-linking by TG appears to be effective only with certain flours due to their specific amino acid composition, the number of exposed glutamine and lysine residues, and the tertiary and quaternary structures present in the proteins (Altindag et al. 2015; Redd et al. 2024). Diowksz and Sadowska (2021) describe using transglutaminase to produce buckwheat bread. Adding the enzyme at 0.05 g/100 g of flour contributed to obtaining a crumb structure with favorable rheological properties, including improved pseudoplastic dough behavior. Furthermore, using the enzyme improved the sensory quality of the final product. The characteristic bitter aftertaste of buckwheat was less noticeable in the experimental bread than in the control sample without TG. Basman et al. (2003) described TG’s effect on baked bread’s characteristics. The study showed that adding 0.25% enzyme resulted in better crumb and crust characteristics, approximately 30% greater loaf volume, and a significant improvement in elasticity compared to untreated bread. The positive effect of TG on bread quality properties was also confirmed by Collar et al. (2005). The authors showed that adding the enzyme at 0.5 mg/100 g of flour resulted in a 31% increase in bread aroma intensity and an 11% increase in bread volume compared to the control sample without transglutaminase. Another study found that adding TG at 3 U/kg of flour to low-wheat doughs significantly improved the quality of the dough and the resulting bakery products (Dube et al. 2007). It is important to note that excessive TG addition can lead to adverse effects, such as increased bread firmness, reduced volume, and extended rising (fermentation) time (Redd et al. 2024). Shin et al. (2010) reported using 0.01% TG to produce gluten-free bread based on rice flour. The enzyme improved the dough structure and shortened the fermentation time by 4–9 min compared to the control. Therefore, the factors influencing the effectiveness of TG are the enzyme dose and the dough fermentation time. In another study, the possibilities of using extruded flours in combination with various proteins and TG doses in the production of gluten-free bread were investigated. Adding TG at a dose of 10 U/g led to a significant increase in crumb hardness and chewiness. Furthermore, the resulting bread exhibited optimal textural and functional properties, meeting the desired quality criteria (Smerdel et al. 2012). According to Moradi et al. (2021), adding 1% TG enzyme improved the texture of breadcrumbs and delayed the staling process of gluten-free bread. In a study conducted by Yildirim et al. (2018), combining TG and protein was more effective in increasing the hardness of a rice flour–based revani dessert than soy protein alone. Using rice protein cross-linked with transglutaminase allows for forming a stable protein network (Chang et al. 2024). Simultaneously, it contributes to improving the product’s nutritional properties. This approach represents a promising strategy in developing modern food products, especially in the gluten-free and functional food segments.

The next direction of transglutaminase use in the baking industry was the effect of this enzyme on the physicochemical properties of whole-wheat dough and pasta during refrigerated storage. The study showed that adding TG resulted in harder pasta than the control samples after 2 days of refrigeration. In the case of dough, adding 1% TG was found to reduce the tensile strength during refrigerated storage (Kang et al. 2021). According to Sakamoto et al. (1996), the addition of TG to pasta improves its strength and acts as a countermeasure against textural deterioration during cooking (Gharibzahedi et al. 2019; Ramos et al. 2023). Another study found that the cooking process did not reduce the hardness or elasticity of oat pasta (Wang et al. 2011). Studies by Xing et al. (2016) showed that the addition of TG during tofu production results in protein modification, increasing the feeling of satiety and reducing the immune system response to soy proteins (Duarte et al. 2019; Yuan et al. 2017).

One interesting and unconventional application of transglutaminase is its use to restore the functionality of the gluten network in wheat flour obtained from grains damaged by insects (Bonet et al. 2005; Caballero et al. 2005). In many countries, many crops do not meet quality requirements due to mechanical damage or environmental stress, preventing their further technological use and leading to raw material losses. Research into the possibility of biotechnological recovery of such crops using enzymatic modification is highly promising for food producers and the agricultural sector, which suffers direct economic losses. Using transglutaminase to modify proteins in damaged wheat flour improves rheological and functional properties, bringing them closer to those typical of high-quality flour (Bonet et al. 2005).

In summary, it can be stated that transglutaminase is a widely used enzyme with a recognized technological function, which in many cases improves the quality and efficiency of food production.

### Dairy industry

Transglutaminase is widely used in the dairy industry (Romeih et al. 2024). Due to its enzymatic properties, it has been used to form strong bonds between various milk proteins (Niu et al. 2025). Cross-linking of milk proteins by mTG leads to the formation of narrow pores between peptide chains, which retain water. Therefore, the new peptide structures that form result in greater firmness and viscosity of dairy products (Marhons et al. 2023). Studies reported by Chen et al. (2022) and Salunke et al. (2022) showed that the addition of mTG at concentrations ranging from 0.1 to 1.0 U/g protein can increase the apparent viscosity of milk gels by 20–60%, depending on the protein composition and processing conditions. Therefore, mTG is used to improve the structure and texture of cheeses (Razeghi and Yazdanpanah 2020). Furthermore, it improves yogurts’ consistency and sensory properties (Lin et al. 2024). These properties of transglutaminase are also important in extending the shelf life of food products (Kolotylo et al. 2024a). The main protein in milk is casein, which readily reacts with TG due to its low tertiary structure, flexibility, and lack of disulfide bonds (Vasić et al. 2023). The most sensitive proteins of the native casein fraction in micelles are κ-caseins, which are located on the surface of the casein micelle (Marhons et al. 2023). According to Bulca et al. (2022), caseins in particular are among the best substrates for mTG among milk proteins. Furthermore, many other proteins, including α-lactalbumin and β-lactoglobulin, are good substrates for TG-catalyzed cross-linking (Yokoyama et al. 2004). Furthermore, Chen et al. (2022) found that TG facilitates the formation of covalent bonds between milk proteins, improving dairy products’ structural integrity and textural stability. According to Güzeler et al. (2024), adding TG resulted in a dense protein network in Hatay cheese. The enzyme acted as a fat substitute and improved the structural properties of the cheeses. In reduced-fat cheese formulations, the application of 0.3–0.5% mTG has been shown to compensate for fat reduction by restoring hardness values to levels comparable with full-fat cheeses, while increasing yield (Gharibzahedi et al. 2018). Similar conclusions were reached by Wang et al. (2024). The authors found that combining lactic acid and TG during processing can significantly improve the textural properties of the resulting gels by induced dissociation of casein micelles and increased covalent cross-linking. Gels formed by TG-treated casein micelles are characterized by high strength (Li and Zhao 2019). According to information provided by Raak and Corredig (2023), mTG activity in milk concentrates is accelerated, and cross-linking can occur within and between casein micelles. In milk protein concentrates (MPC), enzymatic treatment with mTG has been reported to increase tensile strength by 30–50% and reduce protein solubility, which is beneficial for applications requiring structural integrity, such as processed cheese analogues (Salunke et al. 2022). TG can potentially modify the properties of protein concentrates and micellar casein, improving their functionality and the structural properties of milk proteins (Salunke et al. 2022). Studies conducted by the same authors showed that adding mTG alters the melting and tensile properties of protein concentrates and casein micelles by modifying the casein surface through covalent bonds. Studies conducted by Niu et al. (2025) showed that 0.5% mTG significantly accelerated the gelation process and lowered the gel formation temperature of camel casein protein. Furthermore, the authors found that adding TG improved casein gels’ mechanical properties and water retention capacity.

Among other dairy products, yogurt is very popular worldwide (Florowski et al. 2022; Salunke et al. 2022; Niu et al. 2025). Therefore, its production is an important area of dairy product processing using transglutaminase. Adding TG significantly increases the apparent viscosity and improves the water-holding capacity of this product (Chen et al. 2022). The scientific literature contains numerous studies on yogurts obtained enzymatically using TG (Kieliszek and Błażejak 2017). Characteristic properties of these products include their uniformity, firm, creamy consistency, and smooth surface. Research conducted by Bulca et al. (2022) showed that adding 3 U/g TG improved the structural properties of yogurt made from camel milk. Hovjecki et al. (2021) found that mTG positively influenced the physical properties (hardness and whey separation) and the sensory properties of goat milk yogurt. According to Marhons et al. (2023), the addition of TG positively affected the rheological properties of yogurt, including syneresis. Arslan Amin et al. (2023) also demonstrated that syneresis decreased in camel milk yogurt with increasing amounts of the enzyme.

Transglutaminase has also been found to be extensively used in cheese production (El Kiyat et al. 2021). In cheese production, the mTG application (2 U/g protein) has been associated with yield increases, moisture retention improvements, and delayed proteolysis during ripening, particularly in semi-soft cheese varieties (Ceren Akal 2023). Using this enzyme to produce soft cheeses increases their moisture content, palatability, and production efficiency (Romeih and Walker 2017). Research conducted by Domagała et al. (2016) showed that using TG allows for the production of rennet gels with a diverse structure and appearance. According to information presented by Domagała et al. (2022), mTG is not commonly used in producing semi-hard and hard cheeses due to its adverse effect on coagulation. Furthermore, according to the authors, this enzyme prolongs milk coagulation time and negatively affects the gelling properties of the curd. Research presented by Özer et al. (2013) found that combining TG with rennet allows for the production of modified white cheese. The given product was characterized by higher yield, greater hardness, and slower proteolysis than the control cheese. Similar results were obtained by Aaltonen et al. (2014), who modified milk proteins with TG before pasteurizing milk intended for cheese production. They observed an increase in both yield and hardness of the finished product. Aly (2015) concluded that TG increased moisture, the degree of proteolysis, and the total protein content, and improved the organoleptic properties of Gouda cheese. Seyed-Moslemi et al. (2021) demonstrated that mTG caused the formation of inter- and intrapeptide bonds and increased the degree of protein polymerization. These processes increased the protein network’s ability to retain water in the cheese structure.

Transglutaminase is used in the context of ice cream to improve the consistency and stability of dairy products. It has been shown that the addition of mTG (0.1–0.4%) to ice cream increases its fluffiness, slows down the melting rate, and improves the foam stability during storage (Gharibzahedi et al 2018). Ice cream production with this enzyme results in improved aeration and foam stability. Another benefit of using mTG is increased gel strength and improved storage stability. These conclusions were confirmed by research conducted by Kasprzyk et al. (2016). The authors demonstrated the beneficial effect of mTG on ice cream properties. The addition of the enzyme slowed the melting process and allowed the ice cream to maintain its shape for extended periods. Another study (Al et al. 2020) found mTG to be a significant factor in improving the physicochemical properties of ice cream. Simultaneously, the enzyme increased the overflow value of ice cream samples, indicating a more stable protein film. Transglutaminase is an exciting application in producing ice cream with varying fat content (Rossa et al. 2012). The study demonstrated that adding mTG effectively resulted in higher flow, greater fat coalescence, and greater melting resistance of the ice cream compared to samples without mTG. These modifications can be attributed to forming a more cohesive protein network, which increased the stability of the resulting ice cream. The authors also concluded that mTG represents a potential fat substitute in these dairy products.

In summary, using transglutaminase in dairy processing is an innovative approach to improving products’ quality and utility value without compromising their sensory properties.

## Challenges and opportunities associated with the use of transglutaminase

Transglutaminase is a key in biotechnology, particularly in the food industry (Liu et al. 2025). In recent years, scientific research has provided new insights into the challenges and opportunities associated with its use. One of the main challenges requiring special attention is the potential health risks associated with mTG consumption. Literature data indicate (Lerner et al. 2017) that mTG may induce immune responses in people with celiac disease. Furthermore, there are speculations about the impact of mTG on the gut microbiome (Kolotylo et al. 2023). Therefore, further intensive research to assess the safety of mTG use in food is crucial. Another critical issue is the growing consumer awareness of food ingredients with health-promoting properties. More and more people are seeking food products with health-promoting properties without chemical additives. Such information, as well as education and training among consumers and industry employees, can influence the acceptance of this enzyme in food.

**Fig. 4:**
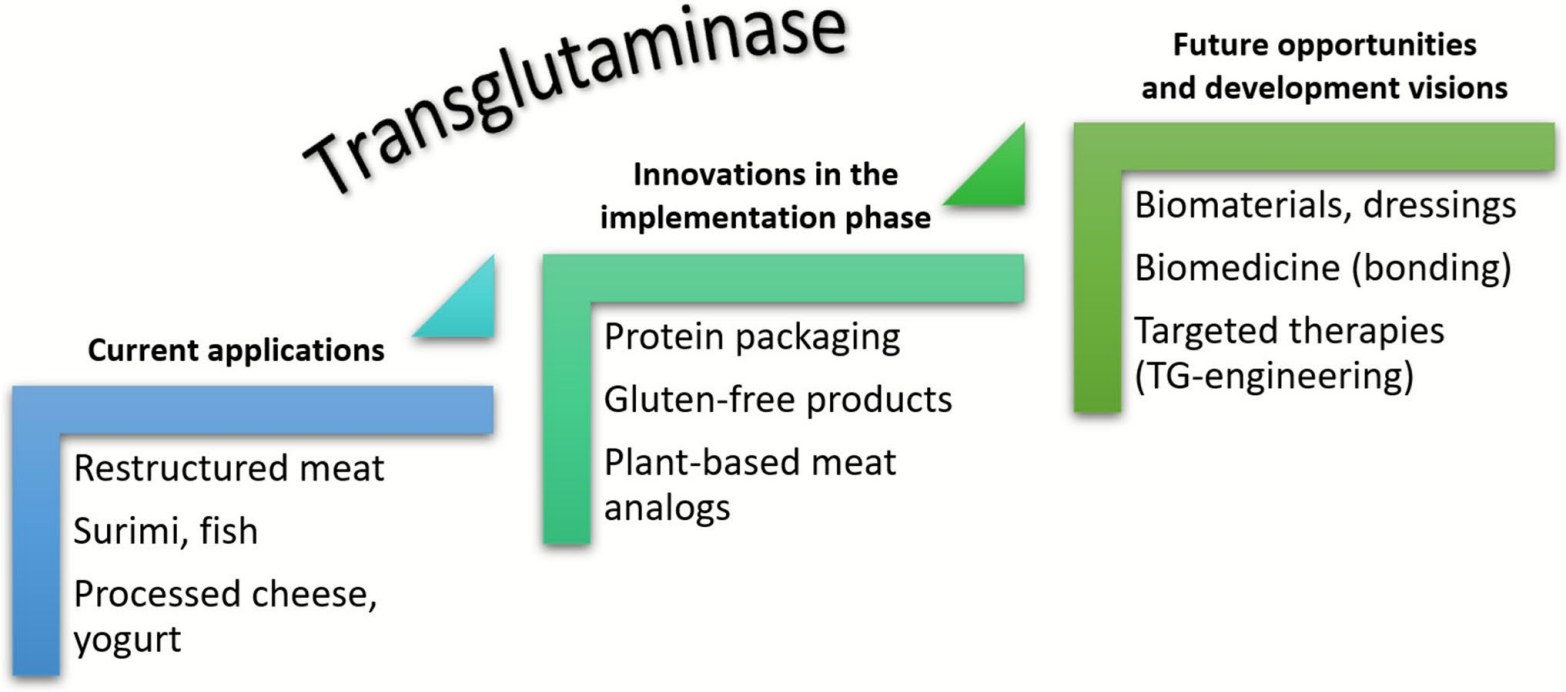
Future perspectives on the use of transglutaminase in the food industry

**Fig. 5:**
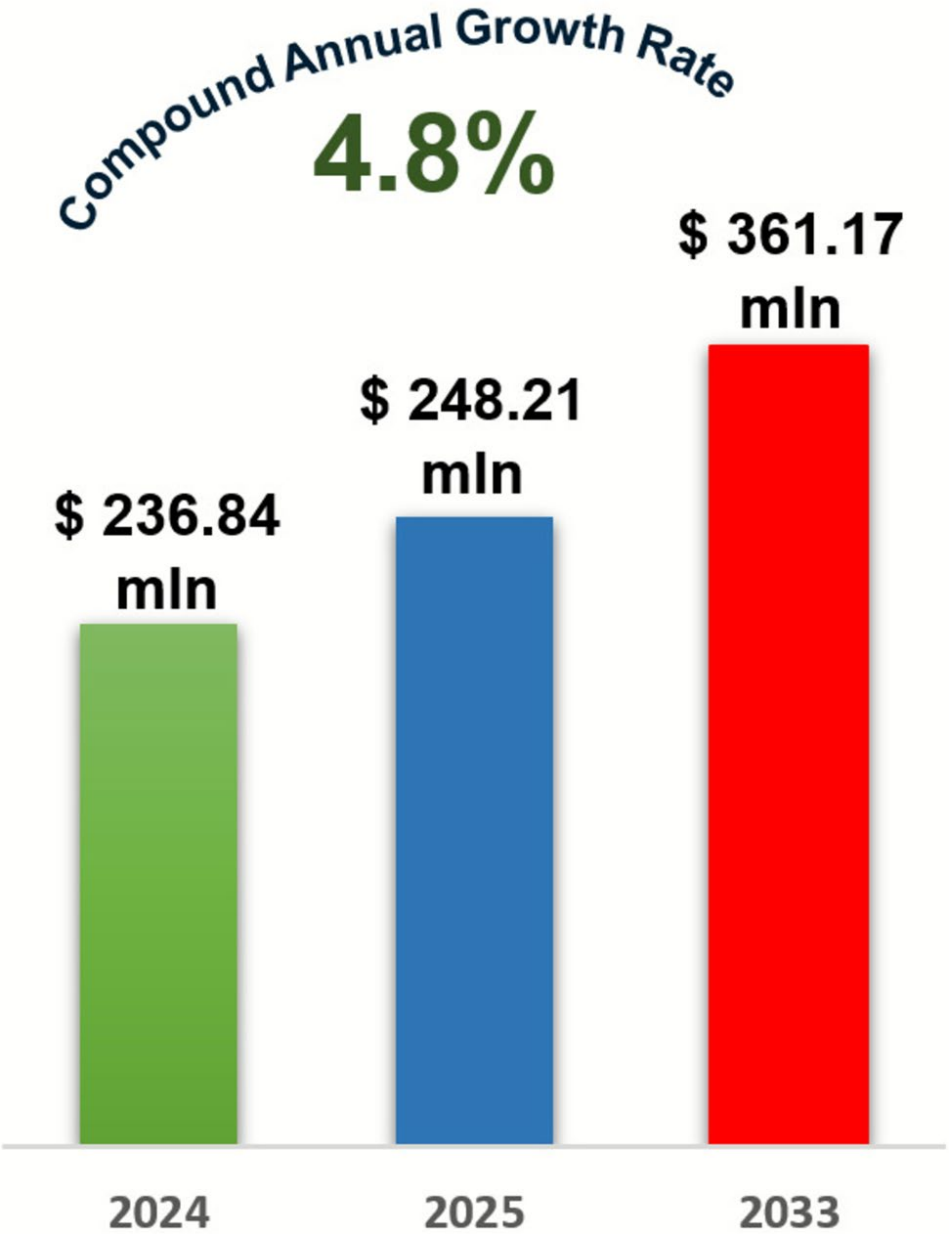
Analysis of the projected value of the transglutaminase enzyme market in the world

It is worth emphasizing that mTG offers significant opportunities for technological innovation in the food industry. Figure 4 shows the potential future applications of microbial transglutaminase (mTG) in industry. Summary information from Global Growth Insights (2025) indicates that TG is gaining popularity. The global market for this enzyme was valued at $236.84 million in 2024 and is projected to reach $284.21 million in 2025. This market is expected to grow to $361.17 million in 2033 (Fig. 5). The information presented demonstrates TG’s potential in creating new food products that meet consumers’ expectations, seeking alternatives to traditional dairy and meat products. It is worth emphasizing that the biotechnological potential of bacterial transglutaminase holds great promise. Research into obtaining different enzyme variants with increased substrate specificity (Tagami et al. 2009) could be crucial for the development of various industries (Suzuki et al. 2024). Advances in protein engineering and computer modeling analyses enable the development of mTG variants selectively targeting specific food proteins, such as plant or dairy proteins. Such structures can be successfully used for controlled cross-linking (Abdalrazeq et al. 2025). Importantly, such processes could significantly reduce the occurrence of undesirable side reactions, as exemplified by the nonspecific deamidation of gliadin peptides (Stamnaes et al. 2008). This is particularly important for products containing gluten. Therefore, focusing design research on computer-based methods could accelerate the development of this field of science. Identifying different mTG variants optimized for industrial conditions, including pH, temperature, and ionic strength, could be a promising direction for further development in this field of science (Granados-Carrera et al. 2025).

Another exciting challenge for industrial development is the use of mTG in creating meat analogues characterized by high sensory quality. This is particularly important in the growing demand for alternative protein sources. Scientific research should focus on using transglutaminase to improve the texture, cohesiveness, and organoleptic properties of meat-like products. Another crucial issue is the use of mTG in the production of bakery and dairy products. It is worth emphasizing that the use of this enzyme is associated with technological challenges, such as optimizing enzymatic reaction conditions and maintaining appropriate enzyme stability under various process conditions. Integrating mTG into production lines is also crucial. Using this enzyme in continuous production allows for the automation and standardization of the protein cross-linking process, translating into consistent quality and reduced production losses for individual food ingredients. Such activities impact the competitiveness of a given food product on the market.

An equally important aspect of future industrial implementation is the control and automation of mTG-catalyzed processes. The use of process analytical technology (PAT) is increasingly recognized as essential for real-time monitoring and control of enzymatic reactions in continuous production systems (Gerzon et al. 2022). Monitoring individual measurements of viscosity, torque, or rheological properties of various products can be an indicator of the progress of protein cross-linking (Tang et al. 2006). Such real-time data enables dynamic adjustment of enzyme dosage and incubation time. These processes will ensure consistent product quality while minimizing overprocessing, enzymatic losses, and production losses. Harnessing the potential of PAT with automated control systems could be a key step toward robust, scalable, and cost-effective use of mTG in industrial biotechnology (Rathore et al. 2010).

It is worth emphasizing that there is growing interest in the biotechnological use of TG outside the food industry (Duarte et al. 2019), specifically in medicine and the production of biomaterials (Rachel and Pelletier 2013). These interdisciplinary applications could further increase the enzyme’s market value and contribute to the emergence of new industries. However, it should be noted that the development prospects for mTG should be closely linked to compliance with clearly defined legal regulations, especially regarding health and safety. These challenges can be addressed through further intensive scientific research, which will expand our knowledge of the characteristics of this enzyme and its properties.

In summary, TG represents a promising area of research and innovation in biotechnology and the food industry. Despite existing challenges related to health safety and consumer acceptance, its potential for creating new products, improving food quality, and meeting the growing demand for plant-based alternatives makes it the subject of intensive research and technological development. It is worth emphasizing that the future of this enzyme in biotechnology will depend on the results of further research on its safety, effectiveness, and consumer acceptance.

## Conclusion

Transglutaminase is an excellent example of an enzyme successfully used in modern food biotechnology. This enzyme allows for the combination of quality, functionality, and industrial efficiency in various products. Although its use is already widespread, further research should focus on fully understanding its long-term effects, particularly in the allergenicity of enzymatically modified proteins and interactions with other food ingredients. Prospects include improving production processes and developing new applications for transglutaminase in functional and nutraceutical foods, which could support the stabilization of bioactive peptides and increase the bioavailability of nutrients for consumers. However, growing concerns about its potential impact on health, particularly in the context of the immune system, the development of allergies, and autoimmune diseases, indicate the need for further independent scientific research. A consumer information policy is also necessary to protect public health and make informed food choices. It should be emphasized that combining advanced enzymatic biochemistry with the needs of the constantly developing food industry will be crucial for fully utilizing the potential of transglutaminase in food biotechnology.

## Data Availability

No data was used for the research described in the article.
